# Effects of paracetamol (acetaminophen) on gene expression and permeability properties of the rat placenta and fetal brain

**DOI:** 10.12688/f1000research.24119.2

**Published:** 2020-08-19

**Authors:** Liam M. Koehn, Yifan Huang, Mark D Habgood, Kai Kysenius, Peter J. Crouch, Katarzyna M Dziegielewska, Norman R Saunders

**Affiliations:** 1 Pharmacology & Therapeutics, University of Melbourne, Parkville, Victoria, 3010, Australia

**Keywords:** placenta, transfer, inflammation, permeability, interleukin-1β, IL1β, α-fetoprotein, AFP, immune response

## Abstract

**Background:** Paracetamol (acetaminophen) is widely used in pregnancy and generally regarded as “safe” by regulatory authorities.

**Methods: **Clinically relevant doses of paracetamol were administered intraperitoneally to pregnant rats twice daily from embryonic day E15 to 19 (chronic) or as a single dose at E19 (acute). Control samples were from un-treated age-matched animals. At E19, rats were anaesthetised, administered a final paracetamol dose, uteruses were opened and fetuses exposed for sample collection. For RNA sequencing, placentas and fetal brains were removed and flash frozen. Fetal and maternal plasma and cerebrospinal fluid were assayed for α-fetoprotein and interleukin 1β (IL1β). Brains were fixed and examined (immunohistochemistry) for plasma protein distribution. Placental permeability to a small molecule ( ^14^C-sucrose) was tested by injection into either mother or individual fetuses; fetal and maternal blood was sampled at regular intervals to 90 minutes.

**Results:** RNA sequencing revealed a large number of genes up- or down-regulated in placentas from acutely or chronically treated animals compared to controls. Most notable was down-regulation of three acute phase plasma proteins (α-fetoprotein, transferrin, transthyretin) in acute and especially chronic experiments and marked up-regulation of immune-related genes, particularly cytokines, again especially in chronically treated dams. IL1β increased in plasma of most fetuses from treated dams but to variable levels and no IL1β was detectable in plasma of control fetuses or any of the dams. Increased placental permeability appeared to be only from fetus to mother for both ^14^C-sucrose and α-fetoprotein, but not in the reverse direction. In the fetal brain, gene regulatory changes were less prominent than in the placenta of treated fetuses and did not involve inflammatory-related genes; there was no evidence of increased blood-brain barrier permeability.

**Conclusion:** Results suggest that paracetamol may induce an immune-inflammatory-like response in placenta and more caution should be exercised in use of paracetamol in pregnancy.

## Abbreviations

AFP, α-fetoprotein; CSF, cerebrospinal fluid; DPM, disintegrations per minute; E, embryonic (note that by longstanding convention all gestational ages in rodents are referred to as embryonic, but in this study E19 is a fetal stage); IL1β, Interleukin 1β cytokine; i.p., intraperitoneal; i.v., intravenous; P, postnatal; RNA-Seq, RNA sequencing; SD, standard deviation; µCi, micro Curie.

## Introduction

Paracetamol (acetaminophen) is commonly taken, either by prescription or self-medication, for the relief of pain and fever. In pregnancy it is the most widely consumed drug, with estimates of expectant mothers talking this medication ranging from 56% in Australia and the Americas ( Wyszynski & Shields, 2016) to 76% in Europe ( Dreyer *et al.*, 2015). The Australian Medicines Handbook (2019) states without qualification that paracetamol is safe for use in pregnancy and breast-feeding. However, epidemiological reports of behavioural effects in the offspring of mothers who took paracetamol during pregnancy are beginning to be published, suggesting a more cautious approach would be appropriate (see Bauer *et al.*, 2018 and Discussion).

In a recent study we have found that paracetamol, when administered to a pregnant dam at doses within the clinical range used in patients, transfers across the placenta to reach the fetus at about 40% of the levels of the drug in the maternal circulation ( Koehn *et al.*, 2019b). Thus, the placenta provides a degree of protection for the developing fetus but the mechanisms involved are not yet understood, nor are the effects that paracetamol may have on placental functions. We have therefore carried out an RNA sequencing (RNA-Seq) study of E19 placentas and brains from control (un-treated) rats and from rats treated with a single (acute) or multiple (chronic) doses of paracetamol. This RNA-Seq study yielded the unexpected outcome of widespread up-regulation of inflammatory and immune-related genes in the placenta of the dam exposed to paracetamol over a prolonged period, with a much less pronounced effect on inflammatory-related genes following a single dose; however, many other genes showed a regulatory response following a single dose of paracetamol. Inflammatory responses during pregnancy have been linked to a range of clinical complications including pre-term birth, fetal cardiac conditions and neurological deficiencies ( Challis *et al.*, 2009; Fleiss *et al.*, 2020; Huleihel *et al.*, 2004; Romero *et al.*, 2007; Salafia *et al.*, 1989). High cytokine levels in blood have been linked to increased blood-brain barrier permeability ( Anthony *et al.*, 1997; Stolp *et al.*, 2005a) and possibly leading to a range of health complications ( Brochu *et al.*, 2011; Nelson *et al.*, 1998; Thornton *et al.*, 2017). Inflammation in the placenta has also been linked to increased placental permeability, as shown in studies that identified a size-dependent increase in maternal-fetal nanoparticle transfer in mice ( Tian *et al.*, 2013).

In the present study, the inflammatory response in the placenta and the fetal brain following maternal paracetamol exposure was examined to see if it was associated with alterations in placental and blood-brain barrier permeability. Placental permeability was assessed using a low-molecular weight, hydrophobic molecule sucrose to determine the transfer in both directions: from the dam’s circulation to the fetal circulation and from the fetal circulation back to the dam. Transfer of a large molecule, the endogenous fetal-derived plasma protein α-fetoprotein (AFP), across the placenta into maternal circulation was also investigated. Results from both of these markers indicate that placental transfer was potentially affected by paracetamol treatment, and demonstrated increased levels of AFP detected in blood plasma of dams treated with paracetamol. The inflammatory cytokine IL-1bß was measured in fetal and maternal plasma; it showed higher levels only in fetal plasma following maternal paracetamol treatment. Permeability of the fetal blood-brain barrier to both small (sucrose) and large (plasma protein) molecules was not affected in spite of increased IL-1ß levels in fetal circulation. The results presented here highlight responses to paracetamol use during pregnancy that appear to be tissue-specific and dependent on duration of treatment. The results are discussed in the context of the appropriate use of paracetamol during pregnancy.

## Methods

### Ethical statement

The animals used in this study were the Sprague Dawley strain of *Rattus norvegicus*. All animal experimentation was approved by the University of Melbourne Animal Ethics Committee (Ethics Permission AEC: 1714344.1) and conducted in compliance with Australian National Health and Medical Research Guidelines. All animals were assessed as healthy prior to commencement of experiments. Animals were monitored prior to and following every injection ensuring there was no abnormalities in weight (>10%), appearance (fur) or behaviour (vocalisation, respiration, movements). All efforts were made to ameliorate any suffering of animals. They were handled by experienced researchers in such a way as to minimise stress prior to being anaesthetised.

### Animals

These were supplied by the University of Melbourne Biological Research Facility and subjected to a 12 hour light/dark cycle with *ad libitum* access to food (dry pellets of a fixed formulation diet for laboratory rats and mice fortified with vitamins and minerals to meet the requirements of breeding animals after the diet is autoclaved or irradiated, supplied by Speciality Feeds, Western Australia) and water. Animals were housed in groups of 2–4 (adult) per cage (25cm x 35cm x 25cm on Breeders Choice paper bedding, made from 99% recycled paper; it is biodegradable with no added chemicals). Age groups investigated (at treatment completion) were embryonic day 19 (E19) pups of both sexes and dams, which were all primigravida 350–400g body weight) and non-pregnant female adults (175–230g body weight). E19 was chosen because this is a stage of development when adequate volumes of blood and cerebrospinal fluid (CSF) can be obtained for analysis from fetal rats without pooling ( Dziegielewska *et al.*, 1981) and individual pups can be injected intraperitoneally while still inside the uterine horn and kept viable for periods of time. Animal numbers were based on previous experience of such experiments and were the minimum number required to detect a significant difference between groups at p <0.05. Animals were selected for treatment groups to ensure weights were statistically similar between direct comparisons. Where possible, equal numbers of male and female fetuses were used. Animals on gestational day E19 were allocated to experiments by animal house staff, who had no knowledge of the particular experiments to be performed. The experimenters had no role in the selection of the animals, thus avoiding selection bias. The numbers (n) of animals used for each experiment are indicated in the relevant Methods or Results section and where appropriate in legends. Two litters in the sucrose permeability studies were excluded from the study. One mother died under anaesthesia. In the other case the fetuses were observed to be in poor physiological state, which would have affected the results.

### Drugs and markers

Paracetamol (acetaminophen ≥99.0%, Sigma-Aldrich) was applied either at a high dose of 15mg/kg (higher limit in the range used clinically, Australian Medicines Handbook, 2019 and Koehn *et al.*, 2019b) or a dose in the lower clinical range of 3.75mg/kg. Paracetamol was dissolved in sterile 0.9% sodium chloride solution for injection. For passive permeability experiments [U- ^14^C]-labelled sucrose (Amersham International, CFB146) was injected in sterile 0.9% sodium chloride solution. Details are described in our previous study ( Koehn *et al.*, 2019b). Estimates of protein (AFP) permeability were obtained from western blot analysis of fetal and maternal plasma, as described below.

### Transcriptomic analysis: RNA-Seq

All experiments took place between 09.00 and 15.00h. Placentas and fetal brains from dams subjected to three treatment regimes were analysed in this study (n=4 for each tissue from each dam).

(i) an E15 pregnant dam was given an intraperitoneal (i.p.) injection twice daily with 15mg/kg of paracetamol (dissolved in sterile 0.9% sodium chloride solution) over four days. On the 5 ^th^ day (E19) the dam was given a final injection of the drug. This experiment is referred to as “chronic”;
(ii) an E19 dam was given a single i.p injection of 15mg/ml paracetamol and is referred to as “acute”; and
(iii) an E19 untreated dam (referred as control).

In experiments (i) and (ii), 30 minutes after the last injection of the drug the tissue samples (placentas, fetal brains) were collected (n=4 for each dam).

For RNA-Seq analysis, placental tissue was sampled as a cross section of the chorio-allantoic placental disc, following removal of the externally attached umbilical and maternal circulatory vessels. Brain samples of the cortex were dissected out as described before ( Koehn *et al.*, 2019a). Samples were collected under RNase free conditions and immediately frozen in liquid nitrogen and transferred to -80 **°**C for storage. RNA extraction was completed using the RNeasy Plus Mini Kits and QIAshredder (Qiagen, catalogue number 74134) for placenta and using the RNeasy Plus Micro Kits (Qiagen, catalogue number 74004) for fetal cortex, following manufacturers specifications. RNA quantity and purity were determined using a NanoDrop ND-1000 UV-VIS spectrophotometer (Thermo Scientific).

RNA samples were transported on dry ice to the Australian Genome Research Facility (AGRF) in Melbourne for Illumina, next-generation sequencing. Runs were 100bp single reads, providing raw FASTAq data. Data were processed using the Galaxy platform and their online software packages ( Afgan *et al.*, 2018). Default parameters were used unless directly specified. Alignment was conducted using HISAT2 (Galaxy version 2.1.0) using the reference genome for rat (rn6; accession number GCA_000001895.4) and the reverse strand setting. For transcript quantification and differential expression analysis, three different methods were employed. In the first, pathway transcripts were assembled with cufflinks (Galaxy version 2.2.1.2) using the reference annotation for rat RefGene (genome) obtained from UCSC Main. Relevant data were passed through Cuffmerge (Galaxy version 2.2.1.1) and analysed for differential expression between groups of interests using Cuffdiff (Galaxy version 2.2.1.5). For the second and third pathway, counts were aligned using HTSeq-counts (Galaxy version 0.9.1) using the reverse strand setting. Generate Count Matrix (Galaxy Australia version 1.0) produced a matrix form of the data, which were then fed through either DEseq2 (Galaxy version 2.11.40.6) or EdgeR (likelihood ratio; Galaxy version 3.24.1) to receive differential expression analysis between treatment groups. Statistically different expression levels between relevant treatment groups were selected if present in two of the three datasets above the statistical threshold of p <0.05 for the adjusted P value of Cuffdiff (Padj), DEseq2 (q value) or EdgeR (FDR). This method of statistical selection minimizes the known false positives and false negatives that can be obtained due to the analysis pathway selected, ensuring all results can be found between multiple pipelines (see Seyednasrollah *et al.*, 2016; Soneson & Delorenzi, 2013). Gene synonym names were produced via bioDBnet ( Mudunuri *et al.*, 2009). Pathway analysis was conducted using DAVID Bioinformatics Resources (version 6.8), with benjamini false discovery rate correction ( Huang *et al.*, 2009a; Huang *et al.*, 2009b).

### Interleukin 1β (IL1β) enzyme-linked immunosorbent assay (ELISA)

IL1β cytokine concentrations in rat plasma were determined using ELISA specific for rat IL1β (R&D systems, Quantikine kit, catalogue number RDSRLB00, monoclonal mouse anti-rat IL1β) following the manufacturer’s protocol. Plasma samples were diluted 1:2 and 50μL of each sample was added to the same volume of assay diluent. Standard dilutions were assayed in duplicate. The plate was incubated at room temperature for two hours, then washed extensively. 100μl of rat IL1β conjugate was added and incubated for a further two hours. After additional thorough washes, the plate was incubated for 30minutes in 100μL of substrate solution then developed with 100μl of stop solution. Plates were read using a FlexStation 3 Multimode Microplate Reader (wavelength 450nm, using 570nm to correct for any optical imperfections in the plate) within 30 minutes of the addition of the stop solution. Cytokine concentrations were determined by comparison with the standard curve produced from each run.

### Permeability across the placenta

All permeability experiments were conducted on E19 dams and fetuses. Two chronic paracetamol treatment regimes were used. Time-mated E15 pregnant dams were injected i.p. twice daily with either a 15mg/kg (referred to as “high”) or 3.75mg/kg (referred to as “low”) dose of paracetamol (dissolved in sterile 0.9% sodium chloride solution) over four days (“chronic” experiments). On the 5 ^th^ day, at E19, these were compared to age-matched animals that were not treated (controls). Numbers (n) of individual experiments are indicated below and are included in the legends of corresponding figures in the Results section.

***^14^C-sucrose permeability.*** Animals were treated either with a “low” dose (3.75mg/kg) or “high” dose (15mg/kg) of paracetamol over four days starting at E15 following the same protocol as above. On the 5 ^th^ day the pregnant dams (E19) were anaesthetised i.p. with 25% w/v urethane, (Sigma, 1ml per 100g body weight) and placed supine on a 35°C heating plate and an endotracheal cannula inserted prior to sampling. Left femoral artery and vein were cannulated. All injections were by slow infusion to the femoral vein; the cannula was flushed with 2ml of heparinized (Hospira Inc, 5000 units per ml) saline. Maternal blood samples were taken from the femoral artery; blood volume was maintained by intraarterial injection of equivalent volumes of 1ml heparinized sodium chloride solution. Blood (right cardiac ventricle), CSF (cisterna magna) and brains (cortex) were sampled from each fetus. Sampling was concluded when the state of the placental circulation (normal condition: umbilical veins pink with oxygenated blood) was deemed insufficient, usually around 90 minutes (see Koehn *et al.*, 2019b for details). CSF samples were examined microscopically for traces of red blood cells and discarded if contaminated ( Habgood *et al.*, 1992). Maternal blood was also collected at the end of the experiment. Blood samples were centrifuged (5000rpm, five minutes). Plasma supernatant was removed and stored at -20°C until used.

Two sets of permeability experiments were conducted:

(i) *Fetal to maternal placental barrier permeability*: pregnant animals treated with paracetamol as described above and control, untreated dams were terminally anaesthetized and an arterial cannula inserted into maternal circulation. Once the uterine horns were exposed, individual fetuses still within their amniotic sacs were injected serially with ^14^C-sucrose as described in Koehn *et al*. (2019b). Each fetus was taken at 30 minutes post injection. Maternal blood samples were collected at the same time as fetuses were consecutively removed for blood sampling. Maternal to fetal plasma levels ratios of ^14^C-sucrose were used as a measure of fetal to maternal placental transfer and calculated as follows:
  $$Fetal\;to\;maternal\;placental\;transfer=\frac{Maternal\;plasma\;at\;time\;y\;(DPM/\mu l)}{Average\;fetal\;plasma\;up\;to\;time\;y\;(DPM/\mu l)}\times 100\%$$
  y=maternalplasmasamplingtime
  One control litter (n=6); one litter from a chronically treated dam with a low dose 3.75mg/kg (n=5) and two litters from two chronically treated dams with a high dose (15mg/kg, n=5 for each litter) were used.
(ii) *Maternal to fetal placental barrier permeability:* pregnant animals treated with paracetamol as described above and control untreated dams were terminally anaesthetized and ^14^C-sucrose was infused into the maternal circulation as detailed for paracetamol permeability studies above. Fetal samples were taken serially between 30 minutes and 90 minutes post injection. Blood samples from individual fetuses were collected together with time-matched maternal blood samples ( Koehn *et al.*, 2019b) and processed for liquid scintillation counting (see below) to obtain fetal/maternal plasma concentration ratios using the equation:
  $$Maternal\;to\;fetal\;placental\;transfer=\frac{fetal\;plasma\;at\;time\;x\;(DPM/\mu l)}{average\;maternal\;plasma\;up\;to\;time\;x\;(DPM/\mu l)}\times 100\%$$
  x=fetalplasmasamplingtime
  One control litter (n=8) and one litter from a chronically treated dam with high dose (15mg/kg, n=6) were used.

***Permeability of a fetal specific protein, AFP- western blotting.*** Levels of AFP in both the maternal blood samples and in fetal samples obtained from experiments of paracetamol treated dams as described above, were estimated using western blotting and antibodies to human AFP (DAKO).

All plasma samples were diluted 10-fold in isotonic saline (0.9%) prior to sample preparation. Samples were run using a total of 9µL of dam and 2µL of diluted fetal sera, denatured in 4x sample buffer (62.2 mM Tris, 5% (v/v) glycerol, 2% (w/v) SDS, and 0.0025% (w/v) bromophenol blue), heated to 95°C for five minutes and centrifuged briefly to remove potential particular matter. 12µL of each sample was loaded onto a 4–12% NuPAGE Novex Bis-Tris Midi gel (Life Technologies) and proteins were resolved at 200V for 40 minutes immersed in MES SDS running buffer (Life Technologies). Gel-resolved proteins were transferred onto PVDF membranes using iBlot gel transfer stacks (iBlot 2; Life Technologies) as per manufacturer's instructions. Membranes were incubated for one hour at room temperature in PBS-T blocking buffer (PBS supplemented with 0.05% (v/v) Tween-20 [Chemsupply]) and 5% (w/v) skim milk powder. Membrane was incubated with AFP primary antibody (AFP, rabbit polyclonal, 1:1000, DAKO, catalogue number A0008, RRID AB_2650473) diluted in the blocking buffer and incubated overnight at 4°C. After three PBS-T washes, the membrane was incubated for two hours at room temperature in horseradish peroxidase-conjugated goat anti-rabbit IgG (Cell Signaling; 1:5000, catalogue number 7074) secondary antibody. Immunoreactive protein bands were visualised by adding 1mL of Enhanced Chemiluminescence mixture (ECL Advance, GE Healthcare) onto membranes and detecting luminescence using a FujiFilm LAS-3000 imager at three and 75 second exposures. Densitometric quantitation of immunoreactivity was performed using ImageJ 2-bit, v1.46 run on OSx 10.14 Mojave on 8-bit TIFF file images. All samples that were directly compared were run on the same gel. Serum from an age-matched non-pregnant female was used as a negative control, while an E19 pregnant dam that was not injected with paracetamol was used as a positive control. Both samples were included on every gel.

### Permeability of the fetal blood-brain barrier

Blood-brain barrier permeability in the fetus was estimated using (i) radioactive sucrose as an example of a small molecular marker permeability and (ii) plasma protein (immunohistochemistry), as an example of a large molecular marker permeability ( Habgood *et al.*, 1993; Johansson *et al.*, 2008; Stolp *et al.*, 2005b). Fetal blood, CSF and brain samples were obtained from the same placental permeability experiments described above.

***^14^C-sucrose permeability.*** For estimation of transfer from mother to fetus, pregnant animals treated with paracetamol (as above) were anaesthetized i.p. with urethane. Starting at 30 minutes after the last maternal injection, embryos were individually extracted. For estimation of transfer from fetal blood to fetal brain and CSF, the fetuses were exposed and injected i.p. with ^14^C-sucrose.

In both types of experiment fetal blood and CSF were sampled as described previously ( Koehn *et al.*, 2019b). Fetal brain samples were taken by opening the cerebral hemispheres to expose the lateral ventricles and a sample of the parietal cortex was removed, taking care to avoid the choroid plexuses. Brain or CSF to plasma ratios of ^14^C-sucrose radioactive counts were used as an estimate of the transfer of sucrose across the blood brain barriers. These were calculated using the equation:

$$Brain\;or\;CSF\;transfer=\frac{Brain\;or\;CSF\;DPM/\mu l}{Plasma\;DPM/\mu l}\times 100\%$$

Treatment groups investigated were control, no paracetamol (n=13), chronic low dose (3.75mg/kg, n=11) and chronic high dose (15mg/kg, n=11) in fetuses that were injected directly. In experiments in which the ^14^C-sucrose was injected into the treated mothers, numbers of pups used were control (n=8), acute (n=10) and chronic high dose (n=6).

***Immunohistochemistry.*** Individual fetal brains were fixed in Bouin’s fixative for 24–48h then dehydrated through graded alcohols, cleared in chloroform and embedded into paraffin wax blocks. These blocks were cut serially into coronal 5µm sections (Leica microtome). Selected sections were heated for 30 minutes (60°C) then washed twice with Histolene (Fronine) for 10 minutes, then five minutes. The sections were rehydrated through graded alcohols for five minutes each (100%, 100%, 95%, 70%) and washed in phosphate buffered saline with 0.2% Tween20 for five minutes. Peroxidase and protein blockers (DAKO) were added to sections and incubated at room temperature for two hours each to block non-specific binding. The primary antibody, plasma protein (anti-rat whole serum, SIGMA, catalogue number R5129, rabbit polyclonal) diluted 1:3000 in a blocker (0.5% fish gelatine and PBS + Tween20), was applied to the slides and incubated overnight at 4°C. After three washes of PBS + Tween20 for five minutes each, the secondary (swine anti-rabbit, DAKO, catalogue number Z0196, polyclonal) and tertiary antibodies (rabbit PAP, SIGMA, catalogue number P1291) both diluted 1:200 were each added and incubated for two hours at room temperature with washes between incubations. Sections were developed with DAB (Diaminobenzidine) using DAKO DAB+ kit (catalogue number K3468) according to manufacturer’s directions and washed in running water for five minutes. Sections were dehydrated through a series of graded alcohols (70%, 95% for five minutes, then 100% for 10 minutes), then 3x five minutes in histolene washes. Slides were then mounted using DPX mounting medium (Fronine). Stained sections were examined under a compound microscope (Olympus, BX50) fitted with a digital camera (Olympus DP70). One control slide was included with every round of immunostaining and had the primary antibody omitted but was otherwise treated in the same way. These were always blank. A total of 11 brains with at least two brains per treatment group were prepared and serially sectioned and mounted on glass slides. Each slide contained 6–8 sections, every 10th slide was stained with haematoxylin and eosin for general morphology. One or two adjacent slides per brain were immunostained for plasma protein from comparable brain regions.

### Liquid scintillation counting

Plasma (10μL), CSF and every injectate (1μL of 1:10 dilution) were weighed and transferred into scintillation vials. In all experiments the radioactivity in the injectate was measured to confirm the uniformity of the injected material. Soluene350 (0.5ml, PerkinElmer) was added to the brain samples and incubated overnight at 36°C. Prior to measurement, two drops of glacial acetic acid (Sigma) were added to brain vials to neutralize the strongly alkaline Soluene350. All samples were mixed with 5ml of scintillation fluid (Emulsifier-safe, PerkinElmer) and measured on the liquid scintillation counter (Tri-Carb 4910 TR, PerkinElmer). Counting was conducted in disintegrations per minute (DPM) for five minutes each with luminescence correction on. Vials containing control, non-radioactive tissues processed identically were also counted simultaneously to establish background counts (which were subtracted from all radioactive samples). Counts were normalized to the sample weight and expressed as DPM per µL or µg of sample. Results are described as concentration ratios, defined as a % of the counts (per µL or µg) in the compartment of interest (brain, CSF, maternal or fetal plasma) divided by the counts (per µL) in the plasma compartment of comparison (see also Koehn *et al.*, 2019b).

### Statistics

RNA-Seq data analysis is detailed above, with significance set at p <0.05. For all other experimentation, statistical differences between treatment groups were determined by unpaired Student t-tests using Prism 6.2 (Graphpad Software Inc) with significance set at p <0.05. We also tested our data using ANOVA followed by Tukey's posthoc test; this approach yielded the same outcomes.

## Results

**Figure 1.**
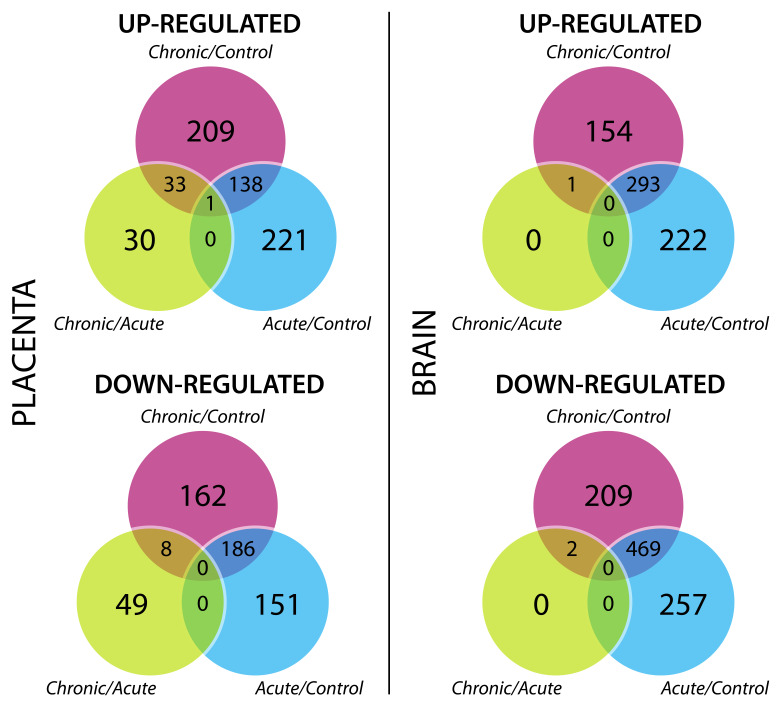
Number of up-regulated and down-regulated genes in the E19 placenta and brain following chronic maternal treatment with paracetamol. Transcript numbers for Chronic/control, Acute/control and Chronic/acute comparisons. Controls were from untreated animals. For details of chronic and acute dosage schedules see Methods. Data derived from RNA-Seq analysis. Overlapping segments represent shared genes.

E19 placentas and brains from three treatment groups were compared for transcriptomic analysis using RNAseq datasets: (i) untreated controls (n=4), (ii) acutely paracetamol treated (n=4) and (iii) chronically paracetamol treated (n=4) dams (see Methods), providing a three-way comparison for each tissue ( Figure 1 and Table 1– Table 5).

**Table 1.** Top 50 up-regulated and down-regulated genes in the E19 placenta following treatment with paracetamol.

	E19 Placenta															
	Up-regulated (acute/control)				Down-regulated (acute/control)				Up-regulated (chronic/control)				Down-regulated (chronic/control)		
	ID	Gene	FC		ID	Gene	FC		ID	Gene	FC		ID	Gene	FC
1	NM_031582	*Aoc3*	4.68		NM_201419	***Clca4***	-54		NM_013025	*Ccl3*	42		NM_012493	***Afp***	-2332
2	NM_001105894	*Chodl*	3.98		NM_012493	***Afp***	-18		NM_053647	*Cxcl2*	23		NM_001085352	***Apoc2***	-1698
3	NM_001164726	*Fcrl6*	3.84		NM_012559	***Fgg***	-18		NM_031512	*Il1b*	13		NM_013162	*Rbp4*	-1415
4	NM_001106063	*LOC290595*	3.45		NM_019373	***Apom***	-18		NM_022194	*Il1rn*	7.75		NM_019287	*Apob*	-900
5	NM_001317798	***LOC684107***	3.37		NM_001085352	***Apoc2***	-16		NM_019282	*Grem1*	5.62		NM_020071	*Fgb*	-639
6	NM_001191752	***Ccdc77***	2.95		NM_053577	***Spp2***	-16		NM_130741	*Lcn2*	3.89		NM_013112	*Apoa2*	-474
7	NM_199233	*Doxl1*	2.84		NM_012738	***Apoa1***	-16		NM_001131001	*Fcer1g*	3.20		NM_012738	***Apoa1***	-452
8	NM_001107071	*Cbx2*	2.66		NM_022924	***F2***	-15		NM_001317798	***LOC684107***	3.12		NM_012681	*Ttr*	-356
9	NM_181378	*Ctsm*	2.57		NM_001013110	***Tf***	-14		NM_001025750	*Plek*	2.99		NM_012559	***Fgg***	-278
10	NM_001109120	*Mboat1*	2.44		NM_001107727	***Mttp***	-12		NM_024145	*Fgr*	2.87		NM_053577	***Spp2***	-249
11	NM_031545	***Nppb***	2.40		NM_022519	***Serpina1***	-12		NM_012711	*Itgam*	2.86		NM_022519	***Serpina1***	-236
12	NM_022846	*Prl8a2*	2.36		NM_013198	***Maob***	-10		NM_001007694	*Ifit3*	2.77		NM_201419	***Clca4***	-166
13	NM_144744	*Adipoq*	2.35		NM_001100690	***Myh14***	-8.80		NM_012523	*Cd53*	2.77		NM_019373	***Apom***	-162
14	NM_001329892	*LOC102557319*	2.31		NM_134432	***Agt***	-8.78		NM_017133	*Thbs4*	2.77		NM_022924	***F2***	-77
15	NM_021580	*Prl8a4*	2.28		NM_012737	***Apoa4***	-7.72		NM_021744	*Cd14*	2.71		NM_001013110	***Tf***	-57
16	NM_001101007	*LOC683313*	2.27		NM_013170	***Gucy2c***	-7.72		NM_130426	*Tnfrsf1b*	2.51		NM_001107727	***Mttp***	-43
17	NM_001025679	*Psg16*	2.27		NM_053332	***Cubn***	-7.69		NM_001122776	***Kcnc4***	2.50		NM_053332	***Cubn***	-28
18	NM_022176	*Prl6a1*	2.25		NM_017097	***Ctsc***	-6.18		NM_031545	***Nppb***	2.36		NM_012737	***Apoa4***	-17
19	NM_001245978	*Frem2*	2.22		NM_019158	***Aqp8***	-5.24		NM_001105720	*Nfkbia*	2.33		NM_013170	***Gucy2c***	-17
20	NM_022667	*Slco2a1*	2.21		NM_001108061	***Amn***	-4.59		NM_001107830	*Lamc3*	2.33		NM_013198	***Maob***	-16
21	NM_022198	***Clcn4***	2.17		NM_001106846	***Cldn2***	-3.52		NM_001100827	***Cenpf***	2.27		NM_001100690	***Myh14***	-15
22	NM_001025641	*Psg29*	2.09		NM_001134516	***Uap1l1***	-3.52		NM_053822	*S100a8*	2.26		NM_134432	***Agt***	-14
23	NM_001009623	***Tnfsf13***	2.07		NM_001108224	*Vil1*	-3.34		NM_020100	*Ramp3*	2.25		NM_001108061	***Amn***	-6.89
24	NM_017036	*Prl4a1*	2.03		NM_012511	*Atp7b*	-3.29		NM_030845	*Cxcl1*	2.21		NM_001170403	*Orai2*	-6.36
25	NM_001122776	***Kcnc4***	2.03		NM_133393	***Lfng***	-3.26		NM_057210	*Sv2a*	2.17		NM_017097	***Ctsc***	-6.06
26	NM_031521	*Ncam1*	1.98		NM_031620	***Lfng***	-3.11		NM_001191752	***Ccdc77***	2.17		NM_130829	*Palm*	-5.39
27	NM_001012072	*Ppp1r3c*	1.97		NM_001109116	*Prr7*	-2.99		NM_001031642	*Serpinb1a*	2.17		NM_133393	***Lfng***	-4.30
28	NM_130779	***Adcy3***	1.96		NM_053802	*Tgfbi*	-2.89		NM_013175	*Scg5*	2.17		NM_001012115	*Creb3l3*	-4.18
29	NM_001127635	*Zfp9*	1.95		NM_001025002	***LOC310926***	-2.82		NM_053587	*S100a9*	2.16		NM_001106846	***Cldn2***	-3.77
30	NM_001191862	*Flnc*	1.95		NM_022533	***Pllp***	-2.82		NM_022198	***Clcn4***	2.14		NM_001134516	***Uap1l1***	-3.50
31	NM_017080	***Hsd11b1***	1.94		NM_001108178	***Pls1***	-2.71		NM_001009623	***Tnfsf13***	2.09		NM_019158	***Aqp8***	-3.40
32	NM_001015011	*Il17f*	1.94		NM_030862	*Marcksl1*	-2.71		NM_001008384	*Rac2*	2.07		NR_131064	***RGD1566401***	-3.35
33	NM_017333	*Ednrb*	1.91		NM_001108599	*Scand1*	-2.59		NM_212525	*Tyrobp*	2.07		NM_031620	***Phgdh***	-3.29
34	NM_001100827	***Cenpf***	1.91		NM_139192	***Scd***	-2.56		NM_001011954	*Cybrd1*	2.03		NM_001037210	***Gipc2***	-3.12
35	NM_001191918	*C1qtnf2*	1.89		NM_024160	*Cyba*	-2.49		NM_017080	***Hsd11b1***	1.97		NM_001014193	***Rsrp1***	-3.07
36	NM_031022	***Cspg4***	1.88		NR_131064	***RGD1566401***	-2.48		NM_022954	*Fat2*	1.97		NM_001025002	***LOC310926***	-3.03
37	NM_017198	*Pak1*	1.87		NM_012862	*Mgp*	-2.47		NM_017051	*Sod2*	1.96		NM_139192	***Scd***	-3.03
38	NM_172073	*Tpbpa*	1.85		NM_001109627	***Epop***	-2.47		NM_001009681	*Oasl*	1.95		NM_022533	***Pllp***	-2.74
39	NM_001191915	*Gpr50*	1.85		NM_001014193	***Rsrp1***	-2.46		NM_130779	***Adcy3***	1.92		NM_139087	***Cgref1***	-2.64
40	NM_001034010	*Tril*	1.79		NM_177927	***Serpinf1***	-2.45		NM_001033691	*Irf7*	1.92		NM_001108178	***Pls1***	-2.63
41	NM_134385	*Prl8a9*	1.79		NM_053565	*Socs3*	-2.45		NM_001107887	*Cd163*	1.92		NM_001047891	*RGD1310507*	-2.63
42	NM_053360	*Sh3kbp1*	1.78		NM_001108971	*Myorg*	-2.38		NM_133624	*Gbp2*	1.91		NM_177927	***Serpinf1***	-2.61
43	NM_030994	*Itga1*	1.78		NM_001170584	*Pex5*	-2.31		NM_012924	*Cd44*	1.89		NM_153736	*Prl3a1*	-2.56
44	NM_001109141	*Kctd15*	1.76		NM_139087	***Cgref1***	-2.31		NM_001007691	*Prss23*	1.87		NM_001008890	*Hbe1*	-2.51
45	NM_001135877	*Taf7l*	1.76		NM_001037210	***Gipc2***	-2.22		NM_031764	*Ddr2*	1.85		NM_030827	*Lrp2*	-2.47
46	NM_001106515	*Fermt1*	1.75		NM_001191974	*Chst13*	-2.20		NM_001134858	*Synm*	1.85		NM_172030	*Entpd2*	-2.29
47	NM_001100984	*Ncf2*	1.75		NM_001037659	*Mpp1*	-2.17		NM_001106420	*Adamts12*	1.83		NM_001109627	***Epop***	-2.27
48	NM_001034932	*C1qtnf6*	1.72		NM_001108552	*Trim2*	-2.16		NM_031022	***Cspg4***	1.83		NM_001014790	*Rarres1*	-2.26
49	NM_001271283	*Golm2*	1.72		NM_001012470	*Irf2bpl*	-2.14		NM_207606	*Kirrel1*	1.83		NM_019231	*Mapk13*	-2.24
50	NM_001106402	*Lhfpl2*	1.71		NM_001107052	*Arl4d*	-2.11		NM_019341	*Rgs5*	1.82		NM_001014088	*Eepd1*	-2.22

Fold change of transcript numbers in placentas treated with paracetamol (chronic, acute or control, n=4 in each group). For details of dosage schedules see *Methods*. Data from RNA-Seq analysis. FC = fold change compared to control (p<0.05, see Methods). Colours indicate genes that were upregulated (green) in both acute and chronic treated animals and downregulated (red) in both acute and chronic treated animals. Note that only 10/50 genes were upregulated following both treatments but 34/50 were downregulated following both treatments.

**Table 2.** Changes in gene regulation in E19 placenta following maternal treatment with paracetamol.

E19 Placenta: immune/inflammation-related genes										
Up-regulated (chronic/control)						Up-regulated (acute/control)				
ID	Gene	Ch / Co	Ac / Co	Ch / Ac		ID	Gene	Ch / Co	Ac / Co	Ch / Ac
NM_013025	Ccl3	42	-	34		NM_013095	Smad3	-	1.61	-
NM_053647	Cxcl2	23	-	17		NM_001025721	Colec12	-	1.59	-
NM_031512	Il1b	13	-	17		NM_001008328	Parp3	-	1.50	-
NM_022194	Il1rn	7.75	-	6.19		NM_017113	Grn	-	1.48	-
NM_130741	Lcn2	3.89	-	3.91		NM_133380	Il4r	-	1.46	0.78
NM_001131001	Fcer1g	3.20	-	3.60		NM_031514	Jak2	-	1.35	-
NM_024145	Fgr	2.87	-	2.17		NM_173328	Lgr4	-	1.32	-
NM_012711	Itgam	2.86	-	2.92		NM_001107754	Traf6	-	1.31	-
NM_021744	Cd14	2.71	-	-		NM_001107063	Cdc42ep4	-	1.28	-
NM_130426	Tnfrsf1b	2.51	-	1.89		NM_001191552	Nsd2	-	1.24	-
NM_053822	S100a8	2.26	-	4.72		NM_001017385	Kdelr1	-	1.22	-
NM_030845	Cxcl1	2.21	-	-		NM_012925	Cd59	-	1.21	-
NM_001031642	Serpinb1a	2.17	-	1.68		NM_001106715	Pum2	-	1.19	-
NM_001008384	Rac2	2.07	-	2.83						
NM_001009681	Oasl	1.95	-	-						
NM_001009689	Cdc42ep2	1.79	-	-						
NM_001134555	C1r	1.71	-	-						
NM_012673	Thy1	1.66	-	-						
NM_001136124	Ifitm3	1.56	-	-						
NM_001013062	Thbs1	1.55	-	-						
NM_001106314	Ifitm1	1.52	-	-						
NM_053535	Enpp1	1.47	-	-						
NM_138881	Rsad2	1.45	-	-						
NM_138844	Unc13d	1.44	-	-						
NM_013016	Sirpa	1.43	-	-						
NM_001166403	RT1-T24-3	1.43	-	-						
NM_001191760	Dock11	1.37	-	1.24						
NM_001271227	Tfe3	1.36	-	-						
NM_001100565	Peli1	1.31	-	-						
NM_001108101	Irak3	1.30	-	1.33						
NM_023092	Myo1c	1.30	-	-						
NM_031140	Vim	1.30	-	-						
NM_017256	Tgfbr3	1.26	-	-						
NM_133624	Gbp2	1.91	-	1.86						
NM_021655	Chga	1.70	-	1.71						
NM_013069	Cd74	1.61	-	1.79						

Down-regulated (chronic/control)						Down-regulated (acute/control)				
ID	Gene	Ch / Co	Ac / Co	Ch / Ac		ID	Gene	Ch / Co	Ac / Co	Ch / Ac
NM_020071	Fgb	-639	-	-		NM_001109535	Rab20	-	-1.93	1.89
NM_013112	Apoa2	-474	-	-		NM_001108207	Tnfrsf21	-	-1.75	-
NM_001007729	Cxcl4	-1.55	-	-		NM_053587	S100a9	2.16	-1.71	3.70
NM_001106095	Lig4	-1.53	-	-		NM_173838	Fzd5	-	-1.53	-
NM_001008322	Shmt2	-1.46	-	-		NM_001025707	Tfeb	-	-1.40	-
NM_030826	Gpx1	-1.39	-	-		NM_012939	Ctsh	-	-1.40	-
NM_001108741	Appl2	-1.26	-	-		NM_001025672	Pspc1	-	-1.34	-
NM_001127390	Washc1	-1.25	-	-		NM_013157	Ass1	-	-1.34	-
NM_001108069	Stk11	-1.23	-	-		NM_001031653	Polr3d	-	-1.23	-
NM_001134974	Trim27	-1.20	-	-		NM_001024967	Tmem106a	-	-1.23	-
						NM_019357	Ezr	-	-1.22	-
						NM_053743	Cdc37	-	-1.18	-

Up-regulated and down-regulated inflammatory and immune-related gene changes in E19 placenta following no treatment (co, controls), acute (ac,) or chronic (ch) maternal paracetamol treatment; see *Methods* for details of dosage schedule. Data from RNA-Seq analysis. Numbers are fold changes for comparisons indicated (Ch/Co, Ac/Co, Ch/Ac). The chronic/acute comparison indicates significant differences in regulation between the two dosage regimes (P<0.05, see Methods). In all cases expression was greater with chronic treatment. This table includes only genes with inflammatory and immune-related functions and thus includes some of the highly expressed genes in Table 1.

**Table 3.** Top 50 up-regulated and down-regulated genes in the E19 fetal brain following paracetamol treatment.

	E19 Brain														
	Up-regulated (chronic/control)				Down-regulated (chronic/control)				Up-regulated (acute/control)				Down-regulated (acute/control)		
	ID	Gene	FC		ID	Gene	FC		ID	Gene	FC		ID	Gene	FC
1	NM_001258011	**Snhg11**	3.41		NM_053304	**Col1a1**	-10		NM_001258011	**Snhg11**	4.13		NM_053304	**Col1a1**	-16
2	NM_012959	**Gfra1**	3.38		NM_001003978	Gspt1	-9.21		NM_001108951	Ak5	3.57		NM_032085	**Col3a1**	-6.99
3	NM_001170531	**Rasgrf1**	2.76		NM_001365151	**Crmp1**	-7.56		NM_012959	**Gfra1**	3.43		NM_001365151	**Crmp1**	-4.70
4	NM_012920	**Camk2a**	2.57		NM_032085	**Col3a1**	-6.98		NM_012920	**Camk2a**	3.28		NM_001008890	**Hbe1**	-3.37
5	NR_037614	Vof16	2.47		NM_001013853	Hba-a3	-5.10		NM_001109003	Cpne4	3.09		NM_033234	**Hbb**	-3.26
6	NM_001105878	Polq	2.30		NM_057212	**Tmem158**	-4.86		NM_012671	Tgfa	3.03		NM_001127523	**Mis18a**	-3.04
7	NM_001105880	**Zbtb20**	2.28		NM_130829	Palm	-4.70		NM_012519	Camk2d	2.94		NM_001109627	**Epop**	-3.02
8	NR_131064	**RGD1566401**	2.27		NM_001025041	**Ier5l**	-3.95		NM_001170531	**Rasgrf1**	2.80		NM_001025041	**Ier5l**	-3.01
9	NM_001191970	**Thsd7a**	2.25		NM_001191905	**Scrt2**	-3.83		NM_001127492	Sphkap	2.73		NM_001108464	**Prr18**	-2.90
10	NM_001191752	**Ccdc77**	2.20		NM_001108599	**Scand1**	-3.72		NM_001191970	**Thsd7a**	2.70		NM_001111269	**Hbb-bs**	-2.81
11	NM_001107310	**Spock3**	2.15		NM_001108464	**Prr18**	-3.71		NR_131064	**RGD1566401**	2.60		NM_001191905	**Scrt2**	-2.74
12	NM_001172305	**Prkcb**	2.12		NM_001109627	**Epop**	-3.24		NM_030993	Ddn	2.59		NM_057212	**Tmem158**	-2.73
13	NR_126581	Lnc215	2.10		NM_001109116	**Prr7**	-3.24		NM_017318	Ptk2b	2.40		NM_001108599	**Scand1**	-2.57
14	NM_021853	**Kcnt1**	2.06		NM_001106014	**RGD1560394**	-3.10		NM_001107671	Plcxd3	2.40		NM_001113223	**LOC100134871**	-2.57
15	NM_001271191	**Sgo2**	2.00		NM_033234	**Hbb**	-3.06		NM_001108335	Rasal1	2.36		NM_001106299	**Ahsp**	-2.57
16	NM_053505	**Slc24a3**	1.98		NM_001111269	**Hbb-bs**	-2.89		NM_001105880	**Zbtb20**	2.34		NM_013197	**Alas**	-2.57
17	NM_206847	**Pfkp**	1.98		NM_030862	**Marcksl1**	-2.62		NM_001271404	Hs6st3	2.34		NR_027324	**H19**	-2.55
18	NM_017007	> **Gad1**	1.98		NM_001107307	**Cilp2**	-2.47		NM_013192	**Kcnj6**	2.32		NM_030862	**Marcksl1**	-2.51
19	NM_013192	**Kcnj6**	1.97		NM_001109270	**Fam110d**	-2.46		NM_001001508	Plppr4	2.28		NM_012651	**Slc4a1**	-2.38
20	NM_053336	Ager	1.97		NM_001008890	**Hbe1**	-2.46		NM_021853	**Kcnt1**	2.26		NM_001109116	**Prr7**	-2.12
21	NR_111959	Miat	1.96		NR_027324	**H19**	-2.37		NM_134363	Slc12a5	2.23		NM_001115013	Selenom	-2.02
22	NM_031003	**Abat**	1.94		NM_001106066	**Pgls**	-2.36		NM_001289778	Map7d2	2.20		NM_181380	**Rtn4rl2**	-2.02
23	NM_001136241	**Ngef**	1.91		NM_001107745	**C1qtnf4**	-2.33		NM_012713	**Prkcb**	2.19		NM_001168650	**Sox12**	-1.99
24	NM_012713	**Prkcb**	1.89		NM_001113223	**LOC100134871**	-2.29		NM_001191752	**Ccdc77**	2.17		NM_001106014	**RGD1560394**	-1.99
25	NR_130129	Tincr	1.85		NM_001168650	**Sox12**	-2.25		NM_001134837	Trps1	2.14		NM_019291	**Car2**	-1.97
26	NM_053817	**Nrxn3**	1.83		NM_013197	**Alas2**	-2.23		NM_030871	Pde1a	2.13		NM_001037659	**Mpp1**	-1.95
27	NM_001202552	Eif4ebp3	1.82		NM_001037659	**Mpp1**	-2.21		NM_053505	**Slc24a3**	2.10		NM_022300	**Basp1**	-1.93
28	NM_001106895	**Tfap2d**	1.82		NM_001164043	**Mapkapk5**	-2.20		NM_001136241	**Ngef**	2.09		NM_001107951	**Dmrta2**	-1.90
29	NM_001014207	Taf1d	1.80		NM_022300	**Basp1**	-2.17		NM_017007	**Gad1**	2.08		NM_001106066	**Pgls**	-1.90
30	NM_024371	Slc6a1	1.79		NM_001109621	**Tpgs1**	-2.14		NM_206847	**Pfkp**	2.07		NM_031114	S100a10	-1.86
31	NM_031783	Nefl	1.78		NM_001107603	**Fbxl15**	-2.11		NM_001135779	Gabra2	2.04		NM_001107307	**Cilp2**	-1.86
32	NM_153630	**Nalcn**	1.75		NM_181380	**Rtn4rl2**	-2.07		NM_031003	**Abat**	2.04		NM_001100741	Col6a2	-1.83
33	NM_001271366	Mki67	1.74		NM_019386	Tgm2	-2.07		NM_001172305	**Prkcb**	2.03		NM_017271	**Nudc**	-1.81
34	NM_017275	**Pnck**	1.74		NM_017271	**Nudc**	-2.03		NM_001108542	Arhgef28	2.03		NM_001109621	**Tpgs1**	-1.77
35	NM_001107659	**Sema5a**	1.72		NM_001071776	Bola1	-2.01		NM_001025664	Wsb1	1.98		NM_001169138	Thbs2	-1.76
36	NM_022631	Wnt5a	1.72		NM_001130570	**Scrt1**	-2.00		NM_001106895	**Tfap2d**	1.98		NM_001164043	**Mapkapk5**	-1.76
37	NM_001107638	Scml4	1.72		NM_001013163	Bmyc	-2.00		NM_173121	Brinp3	1.98		NM_001109270	**Fam110d**	-1.76
38	NM_031046	Itpr2	1.71		NM_001107951	**Dmrta2**	-2.00		NM_001107310	**Spock3**	1.98		NM_001107745	**C1qtnf4**	-1.75
39	NM_001029911	Cit	1.71		NM_031677	**Fhl2**	-1.95		NM_057130	**Hrk**	1.98		NM_001195482	**Ndufaf8**	-1.73
40	NM_031730	Kcnd2	1.70		NM_021678	Camk2n2	-1.95		NM_001107473	Zim1	1.97		NM_001130570	**Scrt1**	-1.72
41	NM_001012235	Impact	1.70		NM_019291	**Car2**	-1.93		NM_017275	**Pnck**	1.96		NM_019354	Ucp2	-1.72
42	NM_057130	**Hrk**	1.69		NM_001134570	**Nupr2**	-1.93		NM_001107881	**Plxnd1**	1.95		NM_031677	**Fhl2**	-1.71
43	NM_001134553	Ubn2	1.67		NM_001195482	**Ndufaf8**	-1.93		NM_177481	Slco3a1	1.95		NM_001025137	Ier5	-1.70
44	NM_133302	Adarb2	1.66		NM_019326	**Neurod2**	-1.87		NM_153630	**Nalcn**	1.93		NM_001108955	Fjx1	-1.70
45	NM_001107881	**Plxnd1**	1.66		NM_001106299	**Ahsp**	-1.87		NM_001271191	**Sgo2**	1.93		NM_019326	**Neurod2**	-1.70
46	NM_052807	Igf1r	1.65		NM_001107574	Znhit2	-1.87		NM_001107659	**Sema5a**	1.92		NM_001107603	**Fbxl15**	-1.69
47	NM_001037139	Pcdhga2	1.64		NM_012651	**Slc4a1**	-1.86		NM_001106498	Pak6	1.91		NM_001107708	Olfml3	-1.67
48	NM_001107201	Prox1	1.64		NM_001127523	**Mis18a**	-1.85		NM_001114656	Jmjd7	1.88		NM_001134570	**Nupr2**	-1.67
49	NM_001107281	Klf12	1.64		NM_012932	Crmp1	-1.84		NM_001108242	Slc9a7	1.87		NM_031838	Rps2	-1.66
50	NM_001107425	Nfat5	1.63		NM_001142652	Neurl1b	-1.82		NM_053817	**Nrxn3**	1.87		NM_019905	Anxa2	-1.66

Fold change of transcript numbers following chronic, acute or control maternal treatment with paracetamol, n=4 in each group. For details of dosage schedules see *Methods*. Data from RNA-Seq analysis. FC = fold change compared to control. Colours indicate genes that were upregulated (green) in both acute and chronic treated animals and downregulated (red) in both acute and chronic treated animals. Note that 26/50 genes were upregulated following both treatments and 40/50 were downregulated following both treatments.

**Table 4.** Changes in gene regulation in E19 fetal brain following maternal treatment with paracetamol.

E19 Brain: immune/inflammation-related genes										
Up-regulated (chronic/control)						Up-regulated (acute/control)				
ID	Gene	Ch / Co	Ac / Co	Ch / Ac		ID	Gene	Ch / Co	Ac / Co	Ch / Ac
NM_053336	*Ager*	1.97	-	-		NM_017318	*Ptk2b*	-	2.40	-
NM_001202552	*Eif4ebp3*	1.82	-	-		NM_001004444	*Zbtb1*	-	1.52	-
NM_022631	*Wnt5a*	1.72	-	-		NM_001108614	*Lime1*	-	1.51	-
NM_001105734	*Dusp10*	1.54	-	-		NM_001107770	*Sppl2a*	-	1.44	-
NM_017187	*Hmgb2*	1.47	-	-		NM_001105733	*Cacnb4*	-	1.31	-
						NM_001107678	*Dhx36*	-	1.16	-

Down-regulated (chronic/control)						Down-regulated (acute/control)				
ID	Gene	Ch / Co	Ac / Co	Ch / Ac		ID	Gene	Ch / Co	Ac / Co	Ch / Ac
NM_001033968	*Bag6*	-1.52	-	-		NM_030858	*Smad7*	-	-1.45	-
NM_001100565	*Peli1*	-1.27	-	-		NM_022701	*Flot1*	-	-1.20	-
NM_001008322	*Shmt2*	-1.21	-	-		NM_017278	*Psma1*	-	-1.17	-
NM_133388	*Rbm14*	-1.19	-	-		NM_001128083	*Trim8*	-	-1.16	-
NM_138710	*Dab2ip*	-1.18	-	-		NM_001106332	*Otub1*	-	-1.16	-
NM_001039914	*Akirin2*	-1.17	-	-		NM_021655	*Chga*	-	-1.14	-
NM_012752	*Cd24*	-1.15	-	-						

Up-regulated and down-regulated inflammatory and immune-related gene changes in the E19 fetal brain following no treatment (Co, controls), acute (Ac,) or chronic (Ch) maternal paracetamol treatment; see *Methods* for details of dosage schedule. Data from RNA-Seq analysis. Numbers are fold changes for comparisons indicated (Ch/Co, Ac/Co; Ch/Ac). Compared to the placenta, in the fetal brain many fewer inflammatory and immune-related genes showed regulatory changes and there were no significant differences between acute and chronic treatments. - indicates no significant difference in fold changes, not that there was no fold change.

**Table 5.** Inflammatory and immune-related gene regulation in both acute and chronic treatment with paracetamol.

E19 Placenta						E19 Brain				
Up-regulated (chronic/control & acute/control)						Up-regulated (chronic/control & acute/control)				
ID	Gene	Ch / Co	Ac / Co	Ch / Ac		ID	Gene	Ch / Co	Ac / Co	Ch / Ac
NM_001009623	*Tnfsf13*	2.09	2.07	-		NM_001172305	*Prkcb*	2.12	2.03	-
NM_001034010	*Tril*	1.63	1.79	-		NM_012713	*Prkcb*	1.89	2.19	-
NM_001033691	*Irf7*	1.92	1.52	-		NM_052807	*Igf1r*	1.65	1.49	-
NM_017269	*Ptprj*	1.70	1.52	-		NM_053374	*Il18bp*	1.60	1.52	-
NM_001106123	*Mrc1*	1.64	1.45	-		NM_001106757	*Cfp*	1.57	1.53	-
NM_019211	*Rasgrp1*	1.45	1.41	-		NM_001276715	*Prkd1*	1.39	1.41	-
NM_001008886	*RT1-S3*	1.31	1.40	-		NM_001079894	*Plekha1*	1.37	1.48	-
NM_019140	*Ptprs*	1.31	1.40	-		NM_001106095	*Lig4*	1.33	1.33	-
NM_012512	*B2m*	1.37	1.37	-		NM_013187	*Plcg1*	1.26	1.24	-
NM_019195	*Cd47*	1.24	1.34	-		NM_012747	*Stat3*	1.24	1.25	-
NM_133395	*Serinc5*	1.26	1.29	-						
NM_052807	*Igf1r*	1.26	1.26	-						

Down-regulated (chronic/control & acute/control)						Down-regulated (chronic/control & acute/control)				
ID	Gene	Ch / Co	Ac / Co	Ch / Ac		ID	Gene	Ch / Co	Ac / Co	Ch / Ac
NM_012738	*Apoa1*	-452	-16	-		NM_032085	*Col3a1*	-6.98	-6.99	-
NM_022924	*F2*	-77	-15	-		NM_001109116	*Prr7*	-3.24	-2.12	-
NM_012737	*Apoa4*	-17	-7.72	-		NM_030826	*Gpx1*	-1.67	-1.52	-
NM_017097	*Ctsc*	-6.06	-6.18	-		NM_030859	*Mdk*	-1.33	-1.39	-
NM_133393	*Lfng*	-4.30	-3.26	-		NM_053761	*Zyx*	-1.33	-1.39	-
NM_001109116	*Prr7*	-2.14	-2.99	-		NM_212509	*Nfkbil1*	-1.43	-1.38	-
NM_024160	*Cyba*	-2.03	-2.49	-		NM_022257	*Masp1*	-1.44	-1.38	-
NM_033351	*Fcgrt*	-1.85	-1.68	-		NM_001277283	*Irak1bp1*	-1.32	-1.36	-
NM_001135922	*Ttll12*	-1.41	-1.54	-		NM_001134974	*Trim27*	-1.40	-1.35	-
NM_001006969	*Irf3*	-1.56	-1.50	-		NM_022546	*Dapk3*	-1.58	-1.35	-
NM_133293	*Gata3*	-1.53	-1.42	-		NM_001106164	*Cmtm3*	-1.40	-1.35	-
NM_001106446	*Zbtb7b*	-1.33	-1.32	-		NM_001108153	*Sema7a*	-1.38	-1.33	-
NM_001025136	*Hexim1*	-1.34	-1.24	-		NM_172045	*Ppp1r14b*	-1.36	-1.32	-
						NM_130411	*Coro1a*	-1.41	-1.31	-
						NM_053669	*Sh2b2*	-1.32	-1.31	-
						NM_001004080	*Gsn*	-1.35	-1.30	-
						NM_053727	*Nfil3*	-1.21	-1.29	-
						NM_012931	*Bcar1*	-1.25	-1.24	-
						NM_001107063	*Cdc42ep4*	-1.26	-1.23	-
						NM_031629	*Psmb4*	-1.26	-1.20	-
						NM_019259	*C1qbp*	-1.20	-1.20	-
						NM_001031653	*Polr3d*	-1.17	-1.19	-
						NM_053743	*Cdc37*	-1.23	-1.19	-
						NM_001047099	*Ythdf2*	-1.18	-1.19	-

Only genes that showed a response in placentas from E19 animals (left panels) and fetal brains (right panels) following both acute and chronic maternal treatment with paracetamol are shown; see *Methods* for details of dosage schedule. Data from RNA-Seq analysis. Numbers are fold changes for comparisons indicated (Ch/Co, Ac/Co; Ch/Ac). There were no significant differences for these genes between acute and chronic treatments, although there were small fold changes (data not shown).

### The effect of paracetamol exposure on placental gene expression (transcriptomic analysis)

As illustrated in Figure 1, following maternal exposure to paracetamol (either acute or chronic), there was a large number of genes that significantly altered their expression in the E19 placentas in two-way comparisons to control tissue, with much fewer that changed between the two treatment groups (chronic/acute). Most genes were uniquely regulated, either up or down, depending on treatment duration, with relatively few that were common to both treatment regimes (64 up-regulated and 57 down-regulated). In contrast, in a three-way comparison, only one gene, *Nfkbia* (NF-kappa-B inhibitor alpha), was shared in all three comparisons ( Figure 1). NFKB is a transcription regulator that is activated by various intra- and extra-cellular stimuli such as cytokines.

The expression of 121 transcripts (the sum of up-regulated and down-regulated genes in the chronic/acute comparison) was significantly different between acute and chronic treatment groups, suggesting an effect of treatment duration. Of these genes, 34 were significantly up-regulated in chronically treated animals when compared to either the acute treatment group or the control group and eight were down-regulated ( Figure 1).

**Figure 2.**
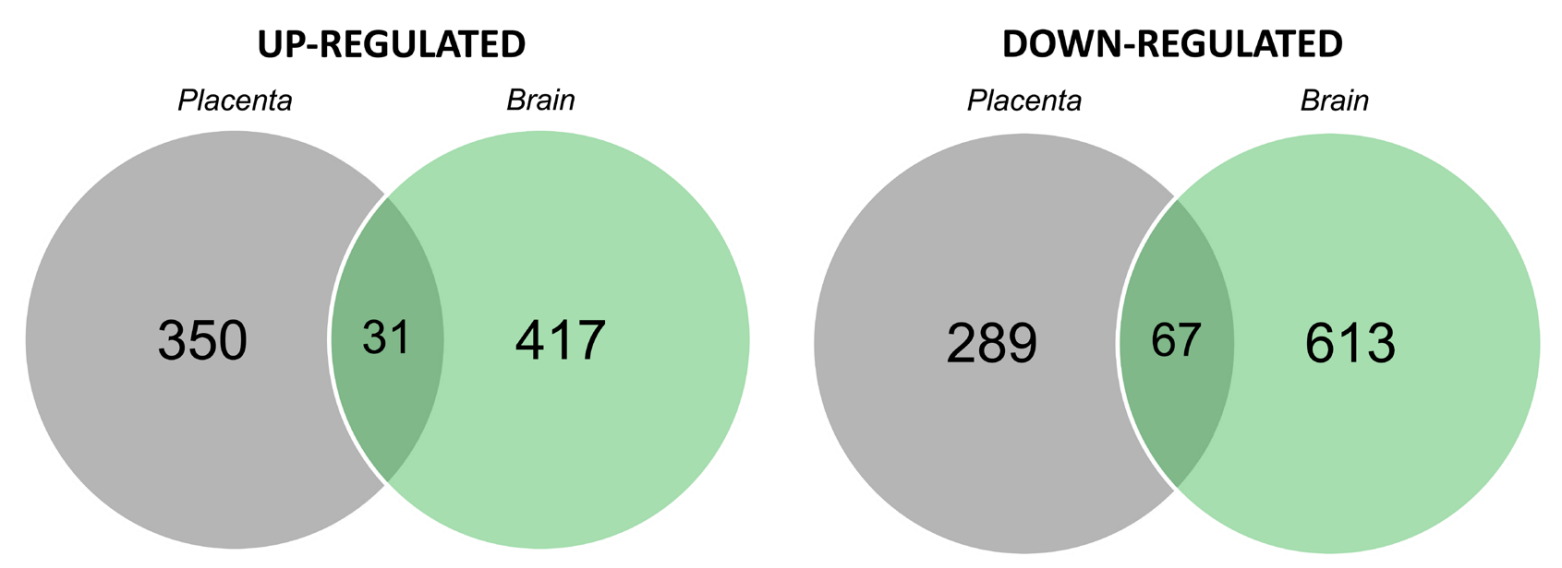
Number of up-regulated and down-regulated genes in the E19 placenta and brain following chronic maternal treatment of paracetamol. Transcript numbers in placenta and brain from chronically (15mg/kg) treated compared to control, untreated animals. For details of chronic dosage schedule see Methods. Data derived from RNA-Seq analysis. Overlapping segments represent shared genes.

Comparing datasets of placentas from chronically treated dams with untreated control dams, the expression of 737 genes was significantly different (either up or down p<0.05, see Methods) ( Figure 2). The top 50 up-regulated and down-regulated genes in E19 placentas are displayed for both acute and chronic treatment groups compared to controls in Table 1. In the E19 placentas, many of the top genes up-regulated following chronic treatment were related to immune-response and inflammation ( Table 1, Table 2 and Table 5). It is difficult to determine the extent to which a statistically difference in gene expression is also functionally significant. It is perhaps worth noting that fewer genes were up-regulated two-fold or more with either acute or chronic paracetamol treatment (25 and 34 genes, respectively) compared to the number that were down-regulated two-fold or more (58 and 61, respectively). In addition, the degree of down-regulation was appreciably greater for many of these genes compared with those that were up-regulated. This was particularly evident for the chronically treated group compared to the control group, with five genes down-regulated greater than 500-fold ( *Afp, apoc2, rbp4, apob and fgb*, Table 1). In addition, 10/50 genes were up-regulated following both treatments but 34/50 were down-regulated following both treatments. Thus overall the down-regulatory effects of paracetamol were much more pronounced than the up-regulatory effects.

Genes that showed a regulatory response in placentas of animals following both acute and chronic treatment with paracetamol are listed in Table 5. Seven of these down-regulated genes showed a fold change of more than two, which was greater in the chronically treated placentas. Other changes were so small that they are unlikely to be of much functional significance.

### The inflammatory response

**Figure 3.**
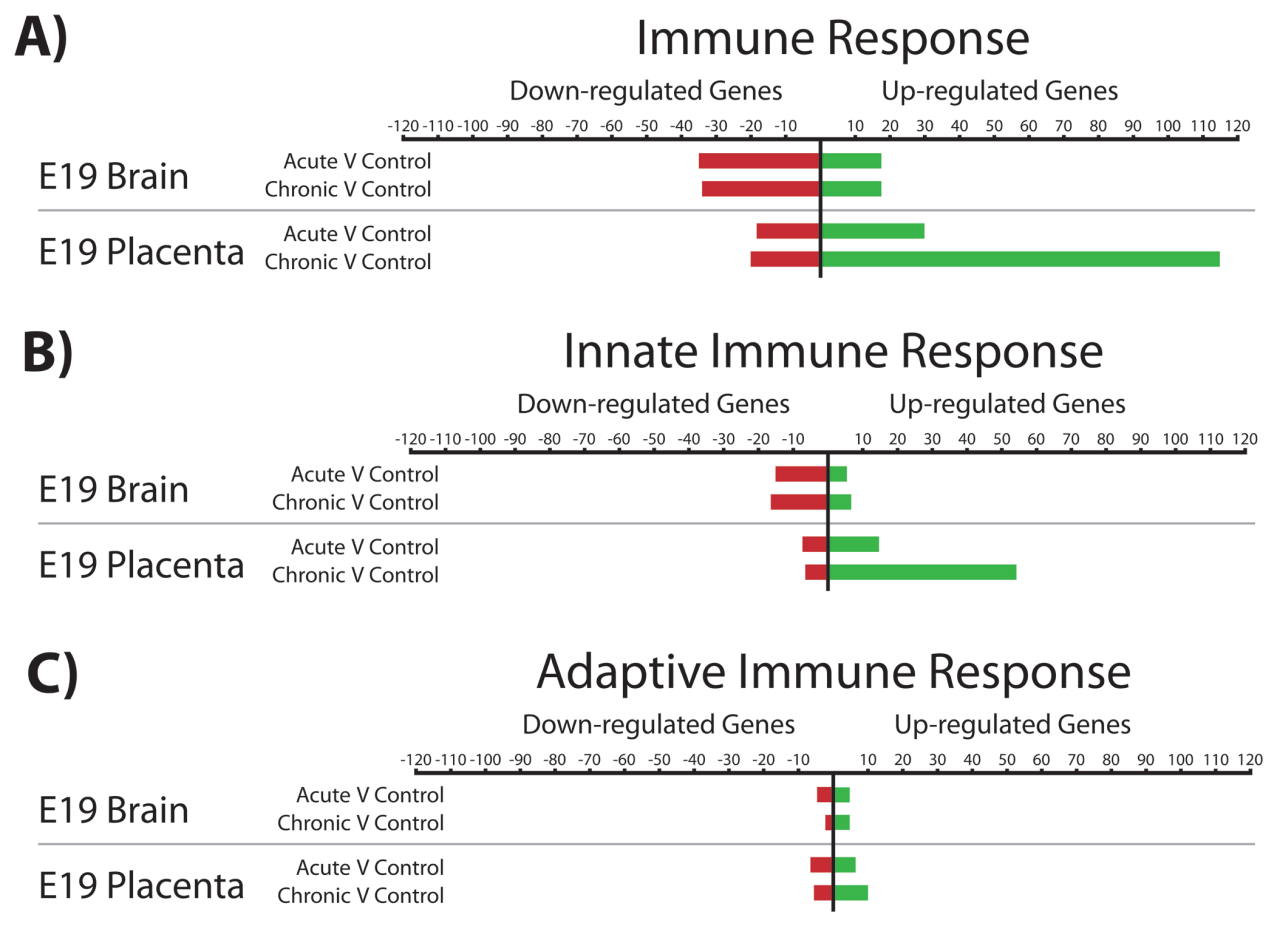
Pathway analysis from the Biological Gene Ontology categories (BP:GO). ( **A**) “immune response”, ( **B**) “innate immune system” and ( **C**) “adaptive immune system”. The number of genes significantly up-regulated (green) and significantly down-regulated (red) are shown for adult brain, E19 brain and E19 placenta, as determined by RNA-Seq. Results are displayed for chronic and acute paracetamol treated rats (n=4). For details of chronic and acute dosage schedules see Methods.

**Figure 4.**
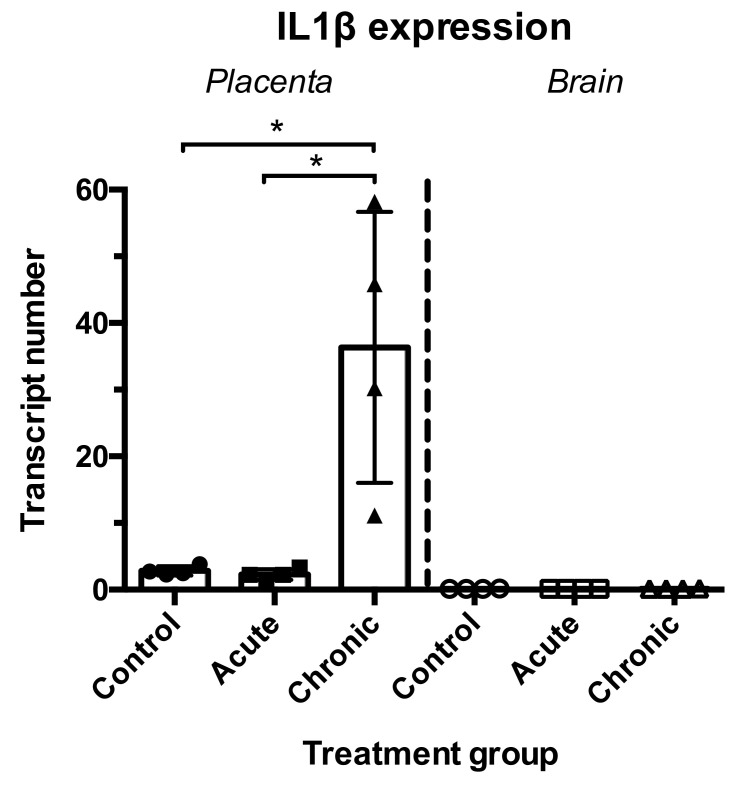
IL1ß transcript counts.

In the placenta of chronically treated rats there was a notable up-regulation of immune response related genes compared to the acutely treated group ( Table 2). Figure 3 illustrates an analysis from biological Gene Ontology (GO) categories of immune response genes (A) subdivided into the innate (B) and adaptive (C) immune systems in the chronically treated animals. In the placenta these included GO biological processes such as neutrophil chemotaxis (p=4.7E-05) and innate immune response (p=0.045). Figure 3 illustrates that the number of significantly up-regulated genes was substantially more than the number of down-regulated genes and that most of these were in the innate immune system category, with a small number in the adaptive immune system. A list of inflammatory and immune-related genes that were up-regulated in the placenta following chronic treatment is shown in Table 2. Overall, some 36 genes showed a statistically significant up-regulation. These included 15 genes that were up-regulated two-fold or more. As can be seen from Table 2, the third most up-regulated gene in the placenta following chronic paracetamol exposure was *Il1ß*. Figure 4 illustrates the number of *Il1ß* gene transcripts in the three treatment groups in the fetal brain and placenta. There was a prominent increase in *Il1ß* transcripts in the placentas from the dams treated with chronic paracetamol and no difference between the datasets of placentas from the control and acutely treated mothers, both showing very low numbers. *IL1ß* is a prototypical marker for inflammation and immune response, with up-regulation in the chronically treated placenta of 13.3 fold change; it could thus be a potential indicator of fetal harm. The response in the placenta following a single acute dose of paracetamol was much more muted ( Table 2).

Transcripts per million in E19 fetal placenta and brain from control (n=4), acute (n=4) or chronically treated dams (n=4) as determined by RNA-Seq. (HTSeq-counts, EdgeR). Means ± SD. * p <0.05.

Amongst the down-regulated genes in the placenta ( Table 1) were several transcripts for plasma proteins (AFP, transthyretin and transferrin, see Discussion) that have been shown to down-regulate under inflammatory conditions (negative acute phase response, Heinrich *et al.*, 1990; Hu *et al.*, 2019; Mackiewicz *et al.*, 1990). Two of these were markedly down-regulated in the acute experiments and further down-regulated in the chronic experiments ( Table 1). This suggest that the response of these plasma protein genes was rapid in onset and continuing over several days in the presence of chronic treatment. In contrast, the up-regulatory response of cytokine genes only became prominent in the placentas of animals chronically exposed to paracetamol ( Table 1; Figure 4).

**Figure 5.**
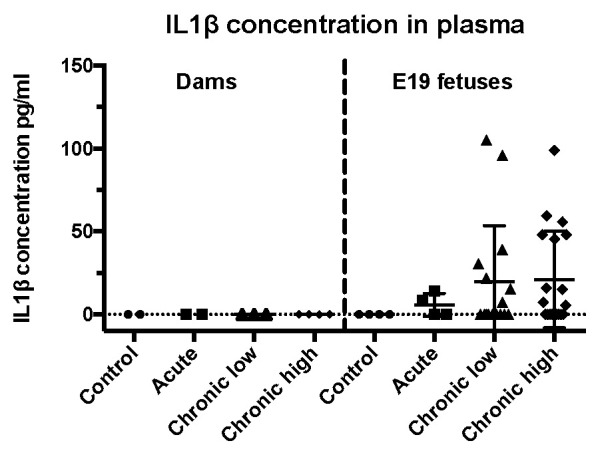
Quantification of IL1β concentration in dam and fetal rat plasma.

***IL1ß concentration (ELISA).*** In order to see if the increase in transcript numbers for *IL1ß* in placentas from dams treated chronically with paracetamol ( Figure 4) translated into an increase in its protein concentration, the levels of this cytokine in plasma of both the dams and pups were measured using a commercially available ELISA kit (see Methods). Results are illustrated in Figure 5. None of the dams in any of the treatment groups had a detectable level of IL1ß in their plasma (limit <5pg/ml) nor was IL1ß detected in the control untreated fetuses. In contrast, IL1ß in the plasma of many of the E19 fetuses whose mothers had been treated with paracetamol was detected. The levels were generally higher in fetuses of mothers treated chronically (acute 2/4, chronic low 7/16 and chronic high 10/19).

Samples from control (4 fetuses from 2 dams), acute (4 fetuses from 4 dams) or chronic paracetamol treated dams at low dose (3.75mg/kg, 16 fetuses from 5 dams) or high dose (15mg/kg, 19 fetuses from 7 dams). Measured by ELISA (R&D Quantikine). Means ± SD.

### The effect of paracetamol exposure on E19 fetal brain gene expression (transcriptomic analysis)

Transcriptomic analysis of the E19 fetal brain was carried out in material collected from the same animals as was prepared for placental analysis, thus allowing a direct comparison between the response of the two tissues to paracetamol treatment of the mother.

As illustrated in Figure 1, following maternal exposure to paracetamol, there was a large number of genes that significantly altered their expression in the fetal brain.

As shown in Figure 2, comparing the dataset for fetal brains from chronically treated dams with untreated control dams, there was a total 1128 genes with significantly different transcript numbers in the E19 brain. The top 50 up-regulated and down-regulated genes in the E19 brain are shown for both acute and chronic treatment groups compared to controls in Table 3. Following both treatments 26/50 genes were up-regulated and 40/50 were down-regulated. Additionally, the level of down-regulation was greater for most transcripts than up-regulation following both acute and chronic paracetamol treatment, for example *Col1a1* (collagen type 1 alpha 1 chain) and *Col3a1* (collagen type 3 alpha 1 chain), see Table 3. There will be a further analysis of the brain data in a later publication ( Koehn *et al., unpublished reports*) that will deal with expression of ABC efflux transporters and related enzymes as these may play a role in the extent to which paracetamol enters the brain at different stages of development ( Koehn *et al.*, 2019b).

### Comparison of the inflammatory response in E19 placenta and brain following maternal paracetamol treatment

In addition to effects of the length of exposure to the drug on gene expression in individual tissues, the regulation in brain and placenta was very different following the same treatment.

Only 98 genes were significantly regulated in both tissues, equating to 5.5% of the transcripts that changed their expression ( Figure 2).

In the E19 placenta many of the top genes up-regulated following chronic treatment were related to immune-response and inflammation, including *Il1ß*, which was 3 ^rd^ highest ( Table 1). In contrast, in the brain, very few transcripts for *Il1ß* ( Figure 4) or other cytokines ( Table 4) could be detected and there was no difference in transcripts for *Il1ß* between the treatment groups ( Figure 4). Table 4 lists the inflammatory and immune-related genes that were up- or down-regulated significantly in the E19 brain. The changes were very small compared to the placenta in both the innate immune and the adaptive immune category ( Figure 3). Table 5 shows immune/inflammatory related genes that showed a regulatory change in both the placenta and the brains from both acutely and chronically treated fetuses.

No changes in plasma protein transcript numbers were detected in the fetal brain (see Discussion). This, together with lack of up-regulation of the inflammatory cytokine *Il1ß*, as seen in the placentas, indicates that an inflammatory response was elicited by paracetamol in the placenta but little or none in the fetal brain. We do not have information if other organs not investigated in this study, such as the liver, could also have been affected.

### Placental permeability

**Figure 6.**
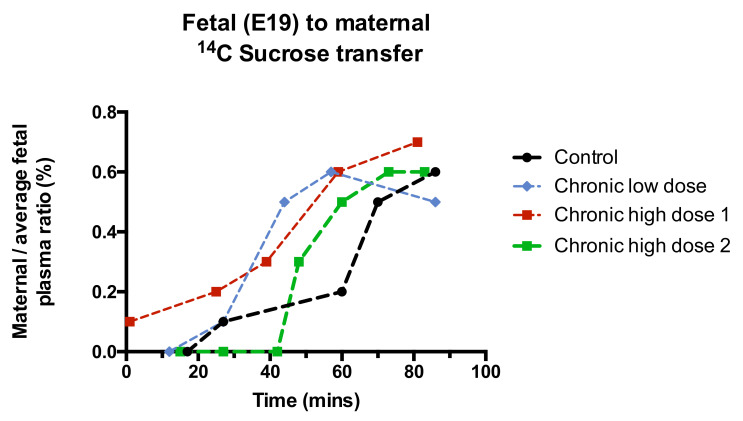
Fetal (E19) to maternal transfer of ^14^C-sucrose. Following maternal paracetamol treatment fetuses, still within their amniotic sacs, were directly injected (i.p.) with ^14^C-sucrose and plasma from both fetus and dam were collected to calculate maternal/average fetal plasma ratio (%) over time. Treatment groups: control (n=6), chronic low dose (3.75mg/kg, n=5) and chronic high dose (15mg/kg, n=5); n refers to number of fetuses. Each data point is a single fetus.

**Figure 7.**
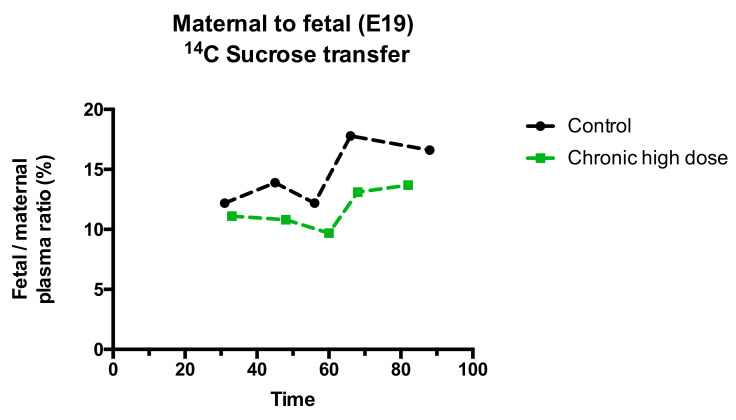
Maternal to fetal (E19) transfer of ^14^C-sucrose. Mothers were treated with paracetamol. ^14^C-sucrose was injected (i.p) into the mothers. Blood samples from individual fetuses were collected at the same time as maternal samples. Treatment groups were: untreated (control, n=8) and paracetamol injected (chronic dose 15mg/kg, n=6) dams. n refers to number of fetuses. Each data point is a single fetus. Transfer calculated as fetal/maternal plasma ratio (%).

In order to investigate if a prolonged exposure to paracetamol and resulting inflammatory response could affect the permeability of the placenta, two sets of permeability experiments were conducted using a small molecular size marker, ^14^C-sucrose (see Methods). These were designed to examine the transfer from the mother to the fetus but also from the fetus back to the mother. Results are illustrated in Figure 6 and Figure 7.

***Fetal to maternal transfer of ^14^C-sucrose.*** To investigate the placental transfer of sucrose from fetus back to the dam following maternal paracetamol exposure, sucrose was injected directly into the pups still within their amniotic sacs (see Methods). Two litters were injected in mothers that had been treated with chronic high doses of paracetamol and one litter from a mother treated with chronic low dose paracetamol. These were compared with one litter from an untreated control mother. Plasma samples from both the fetuses and dam were collected and ratio of ^14^C- sucrose estimated (see Methods). The results are shown in Figure 6. All three of the litters from mothers treated with chronic paracetamol (either high or low dose) showed slightly higher permeability from the fetus back to the mother than in the control dam. However, the ratios are extremely low, making accurate comparison difficult.

***Maternal to fetal transfer of ^14^C-sucrose.*** In order to investigate if the rate of transfer of a small molecular marker from dam to fetus across the placental barrier was affected following paracetamol exposure, dams either untreated (control) or treated with chronic high (15mg/kg) doses of paracetamol were given a final intravenous (i.v.) injection of ^14^C-sucrose 30 minutes before removing their fetuses ( Figure 7). Blood samples from dams were time matched to the removal and blood collection from each fetus (see Koehn *et al.*, 2019b). The transfer from the mother to the fetus in the paracetamol treated dams was slightly less than that in the control animal. The much higher ratios obtained in the maternal to fetal transfer experiments ( Figure 7) compared to the fetal to maternal transfer ( Figure 6) are due to the differences in volume of distribution, hence dilution of the marker, when sucrose is injected into the mother or into the fetuses.

**Figure 8.**
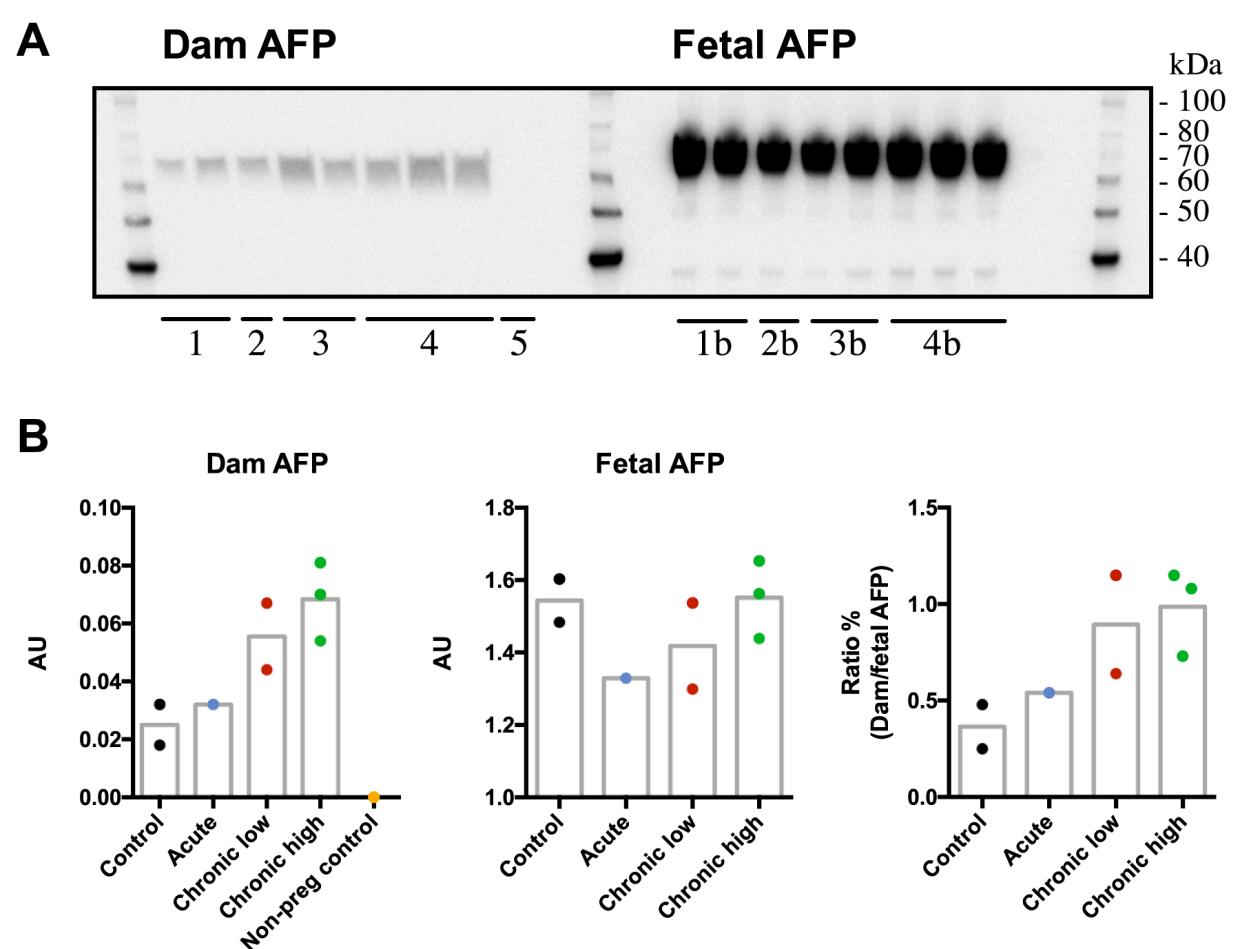
Estimations of α-fetoprotein (AFP) concentrations in fetuses (E19) and dams. **A)** Western blots of AFP in plasma from dams and fetuses in different treatment groups. Numbers for dam blots are samples from individual animals; numbers in fetal plasma blots indicate individual fetuses from corresponding dams. Treatment groups were: control (1/1b; n=2), acute (2/2b; n=1), chronic low dose (3/3b; n=2), chronic high dose (4/4b; n=3) and non-pregnant control (5; n=1). **B)** Estimations of AFP in dam and fetal plasma (densitometry units from blots in ( **A**) and fetal to maternal transfer of AFP expressed as dam/plasma AFP ratio (%). Note: each point represents an individual animal. Note that all chronic treated dams had higher plasma levels of AFP than the un-treated control pregnant dams; AU are ordinate arbitrary densitometry units.

***Detection of AFP in fetal and maternal plasma.*** In order to investigate if exposure to paracetamol can also influence the transfer of a protein from the fetal circulation into the maternal blood across the placenta, western blot analysis was made of fetal and maternal plasma samples using cross-reacting antibodies specific for AFP (see Methods). Figure 8A shows the blot that contained both the fetal and maternal samples together with one negative control (non-pregnant female rat). Densitometry measurements are illustrated in Figure 8B together with maternal/fetal ratios. There was no detectable band in the non-pregnant control sample and all maternal samples showed a much lower level of the protein than fetal samples. The levels of the protein in fetal samples did not appear to change between the control and any of the treatment groups ( Figure 8B), but in maternal samples, AFP levels were higher in all chronically treated dams compared to un-treated controls. This was reflected in the ratios of AFP in maternal to fetal plasma ( Figure 8B, right panel) in which all of the chronically treated animals had ratios that were above those in untreated controls and in one acutely treated animal. Prolonged exposure to the drug increased AFP transfer from fetus to dam by about three times compared to the control animals.

### Permeability of the fetal blood brain barrier

Two different molecular size markers ( ^14^C-sucrose and plasma proteins) were used to assess any changes in blood-brain barrier permeability following chronic paracetamol treatment of the dam. The samples were obtained from the same experiments as the placental permeability studies.

**Figure 9.**
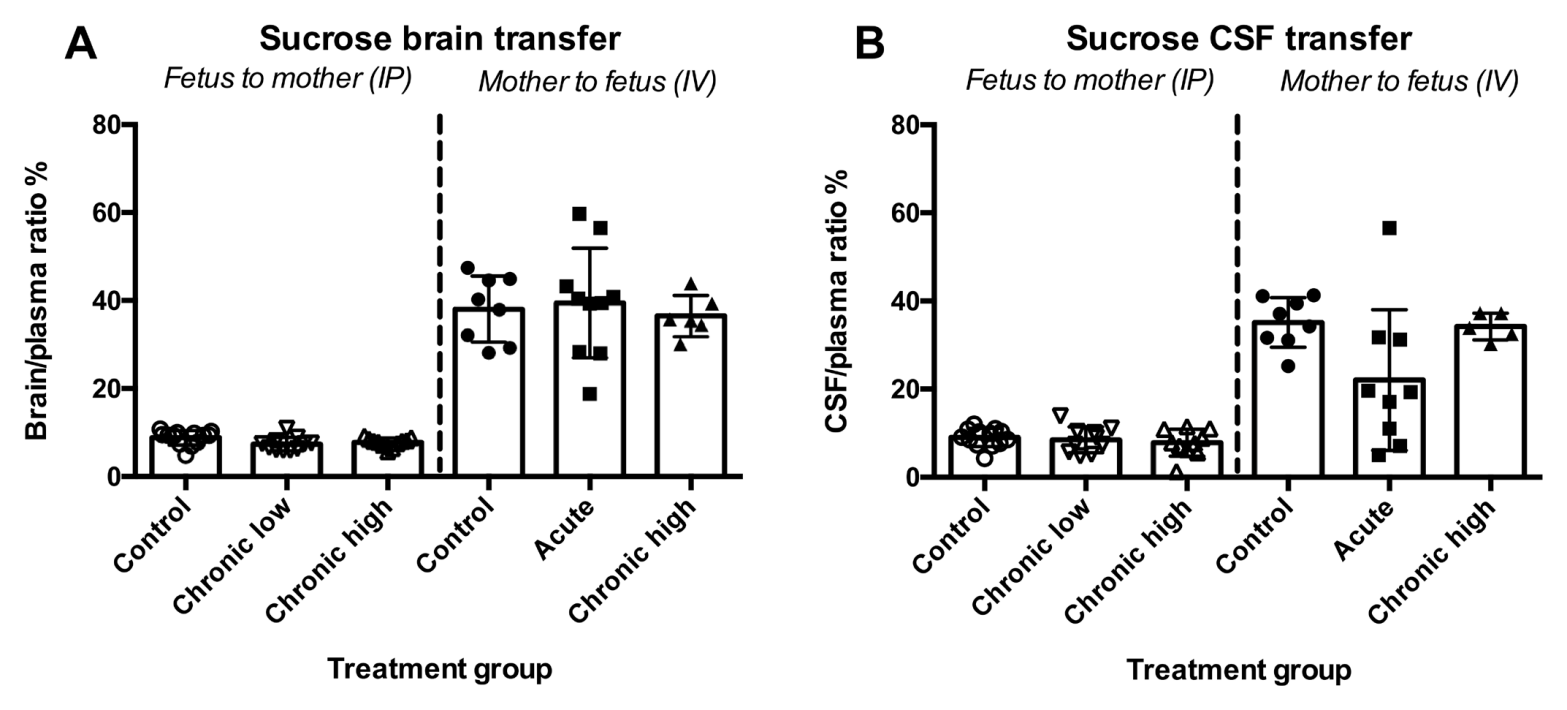
Transfer of ^14^C-sucrose into the E19 brain (A) and cerebrospinal fluid (CSF; B) following paracetamol treatment.

***Transfer of ^14^C-sucrose into the brain and CSF following different paracetamol treatment regimes.*** To investigate the transfer of ^14^C-sucrose into the fetal brain after maternal paracetamol exposure, fetal blood, brain and CSF samples from dams untreated (control) or treated acutely or chronically with either low (3.75mg/kg) or high (15mg/kg) doses of paracetamol were measured. As shown in Figure 9, there was no significant difference in the transfer into the brain and CSF between any treatment groups ( Figure 9).

Fetuses were exposed to ^14^C-sucrose either directly (fetal i.p. injection) or indirectly (maternal i.v. injection). Treatment groups investigated were control, no paracetamol (n=13), chronic low dose (3.75mg/kg, n=11) and chronic high dose (15mg/kg, n=11) in fetuses that were injected directly. In experiments in which the ^14^C-sucrose was injected into the mothers; n numbers were control (n=8), acute (n=10) and chronic high dose (n=6) in the mothers. Means ± SD.

Entry into the brain and CSF when the ^14^C-sucrose was injected directly into the fetus was also investigated and results are illustrated in Figure 9. Here too there were no significant differences in the entry of sucrose into brain and CSF between the three treatments.

However, the fetuses that were directly exposed to sucrose (i.p injection) showed a lower level of transfer into the brain and CSF compared to those that were exposed indirectly (i.v. injection to dam), around 10% compared to 40%, respectively. This reflects differences in distribution volume following the different routes of injection as well as the time involved in samples collection.

**Figure 10.**
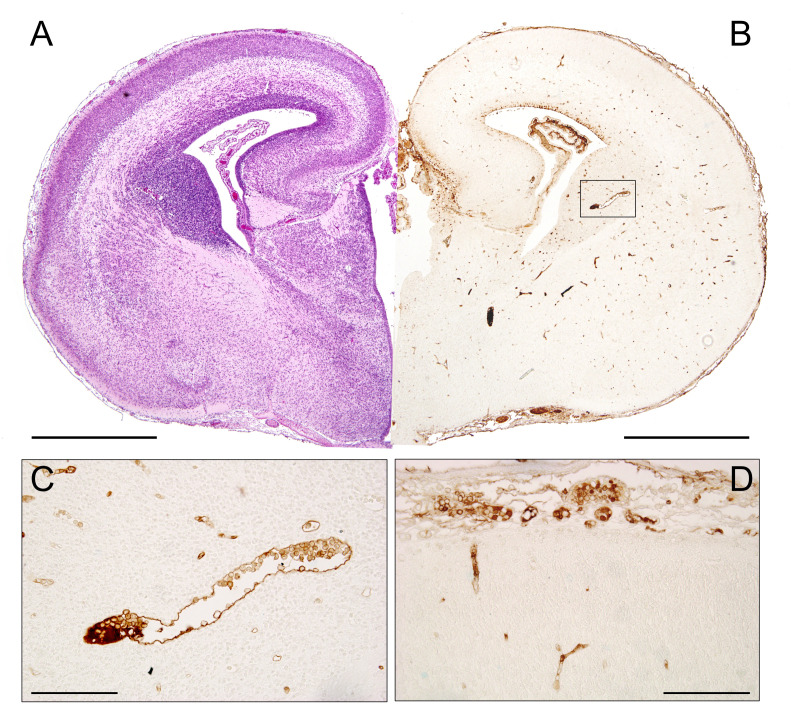
Histology of E19 fetal brains. **A**) Hematoxylin and eosin coronal section of E19 neocortex of fetus from mother treated with chronic high dose paracetamol. **B**) Adjacent section from same brain as **A** immunostained for plasma proteins. **C**) High power image from **B** (box). **D**) High power immunostained image of E19 neocortex of fetus from mother treated with acute high dose paracetamol. Note that all cerebral vessels appear intact with protein immunostained deposits all within blood vessel lumen, indicating that paracetamol treatment has not affected their barrier permeability to plasma proteins. Bars in **A** & **B** are 1mm; bars in **C** & **D** are 100µm.

***Blood brain barrier integrity for endogenous plasma protein.*** Transfer of large molecule plasma proteins into the fetal brain following maternal paracetamol exposure was studied using immunohistochemistry and antibodies to rat serum proteins (see Methods). Brains were matched with plasma samples containing detectable IL1ß levels as estimated by ELISA ( Figure 5). The distribution of the proteins in E19 brains from control, acute and chronic high dose (15mg/kg) paracetamol treated dams is illustrated in micrographs in Figure 10. There was no evidence of a “leak” of protein in any of the vessels in the fetal brains examined. In all sections stained from all brains investigated, immunostaining was exclusively localised in the blood vessels, choroid plexus stroma and precipitated CSF and there was no visible difference in the brain morphology between treatment groups.

Thus, the results clearly show that the blood-brain barrier, at least to plasma proteins and to sucrose, was not affected by paracetamol exposure of the dam, the inflammatory response in the placenta nor the increased levels on IL1ß in fetal blood.

## Discussion

In order for a drug taken by a pregnant mother to reach the fetal brain it has to cross both the placental and the blood-brain barriers. Any changes to normal functioning of these interfaces could have detrimental effects on fetal health and pregnancy outcomes. We have therefore analysed the transcriptomic changes in rat E19 placentas and brains following paracetamol treatment of the dams. Paracetamol is one of the most commonly used medications in pregnancy ( Dreyer *et al.*, 2015; Wyszynski & Shields, 2016). Pregnant rats were treated with paracetamol acutely and chronically and compared to controls (no treatment). The doses used were within the clinically recommended range (0.5g to 4g in 24 hours in adults). In the case of the chronic treatment, this corresponded to a relatively prolonged period of pregnancy in the rat (E15-19, about 25% of gestation). This was followed by investigating placental transfer of small and large molecules from the dam to the fetus and from the fetus back to the maternal circulation, to see whether paracetamol exposure altered barrier function. Finally, the permeability of the blood-brain barrier was analysed in the fetuses of paracetamol treated and untreated dams.

From the results it was apparent that some form of acute phase response was elicited as transcripts for several plasma proteins were down-regulated in placentas of both acute and chronic treated animals ( Table 1). These proteins were AFP, transthyretin and transferrin ( Vranckx *et al.*, 1989), fibrinogen beta chain ( Birch & Schreiber, 1986) and apolipoproteins Apoa1-4, several of which are known to respond to inflammation as negative acute phase proteins ( Tu *et al.*, 1987). Since a marked response was already apparent after a single dose of paracetamol, it seems that this was a rapid response to paracetamol, which was sustained and increased when the treatment was chronic. A summary of transcript numbers for AFP, transferrin and transthyretin, together with numbers for *Il1ß* for comparison, is presented in Table 6 for both the brain and the placenta. These clearly show that some form of acute phase response was taking place in the placenta following paracetamol treatment; however, other typical acute phase response-related cytokines were not up-regulated (such as TNFα or IL6). Transcript numbers in the brain did not change, demonstrating that the acute phase response was tissue specific and restricted to the placenta.

**Table 6.** Transcript numbers in E19 placenta and fetal brain for negative acute phase plasma proteins and IL1β.

E19 placenta												
	Control				Acute				Chronic			
Sample	Afp	Tf	Ttr	IL1β	Afp	Tf	Ttr	IL1β	Afp	Tf	Ttr	IL1β
1	10372	2906	783	2.7	3.5	31	0.7	1.3	2.7	35	1.4	11
2	16873	3789	1100	2.5	3.0	35	2.6	2.2	3.5	28	1.6	30
3	1263	366	65	2.3	1571	413	95	3.3	3.5	17	1.7	46
4	72	56	4.6	3.8	8.4	29	1.6	2.2	2.9	45	1.1	58
E19 brain												
	Control				Acute				Chronic			
Sample	Afp	Tf	Ttr	IL1β	Afp	Tf	Ttr	IL1β	Afp	Tf	Ttr	IL1β
1	0.4	2.8	0.2	0.1	0.2	1.2	2.3	0.1	0.2	2.9	0.8	0.2
2	1.7	2.1	0.5	0.1	0.1	1.2	0.4	0.1	0.1	3.2	1.1	0.1
3	0.2	5.0	0.4	0.2	0.1	1.9	0.2	0.1	0.1	3.5	0.9	0.1
4	0.1	3.3	0.2	0.1	0.2	1.4	0.3	0.1	0.2	2.8	1.0	0.1

In placental samples transcript counts per million for the three negative acute phase proteins were smaller in chronically treated animals compared to controls and all IL1β numbers were greater than in controls. There was some variation in values between individual placentas which was more obvious in samples from acutely treated animal indicating that the response was potentially time-dependent. In all brain samples the transcript numbers were very low, with no evidence of an inflammatory response. This indicates that the response in the placenta was tissue specific.

Several immune and inflammatory-related genes were up-regulated in placentas of animals treated with the chronic dose regime, but much less so in the placentas of acutely treated animals ( Table 1, Table 2 and Table 5). The key inflammatory cytokine, IL1ß, was shown to be present in the blood of a high proportion of fetuses of mothers exposed to both acute and chronic treatment with paracetamol. The levels were variable in different fetuses but generally higher in the chronically treated animals. No IL1ß could be detected in either the maternal blood of paracetamol treated animals or in fetuses of control untreated animals. This confirms that paracetamol was indeed eliciting an inflammatory response but only on the fetal side of the placental circulation. Thiele *et al*. (2015) reported that pregnant mice treated with either 50 or 250mg/kg paracetamol showed some immune responses in the uterus and some morphological changes in the placenta. However, they did not investigate possible immune responses in the placenta and the doses of paracetamol were much larger than the ones we used and were well above the clinical range.

In order to determine if prolonged paracetamol exposure of the dam could affect some aspect of placental function, we have estimated placental permeability to a small molecular marker, sucrose and to large plasma protein AFP in both directions i.e. from the dam to the fetuses and from the fetuses back to the dam. The results showed that there was a small and variable increase in permeability to ^14^C-sucrose and of AFP permeability in the direction from fetus to mother ( Figure 6 and Figure 8). There may also have been a small decrease in sucrose permeability from mother to fetus ( Figure 7) but due to small numbers this is inconclusive.

Placental inflammation induced by lipopolysaccharide (LPS) injection in pregnant rats has been reported to induce maternal serum and placental cytokines and increased maternal serum AFP ( Hu *et al.*, 2019). In those experiments LPS did not increase the expression of AFP in fetal liver, maternal liver or placenta, but did reduce the fetal serum AFP levels, a pattern implying a possibility of increased transfer of AFP from the fetus to the mother, thus depleting it from fetal circulation. We did not find any difference in fetal AFP levels but this discrepancy could be due to either the duration and severity of the response or sensitivity of the methods used.

Permeability of the fetal blood-brain barrier to both sucrose and plasma protein was also investigated. In contrast to the placenta, there was no evidence of a change in brain barrier permeability to either marker in fetuses of dams treated with paracetamol. This is relevant to earlier studies in which inflammation induced by LPS was shown to result in a breakdown of the blood-brain barrier that was age-dependant ( Stolp *et al.*, 2005a; Stolp *et al.*, 2005b). However, it is likely that E19 is at a developmental stage when the response to LPS is not yet developed, as shown in a similar study in a marsupial species, *Monodelphis domestica* ( Stolp *et al.*, 2005a).

### Limitations of the study

The study has been carried out in pregnant rats at a single gestational age (E19). This stage of brain development in rats at E19 is approximately equivalent to 22–24 weeks gestation in humans ( Clancy *et al.*, 2001), corresponding to the earliest age of viability ( Fischer *et al.*, 2009; Stoll *et al.*, 2010). The rat and human placentas are both classed as hemochorial ( Blood *et al.*, 2007; Dawe *et al.*, 2007) but there are differences in morphology, in particular that the rat placenta has more morphological layers between the fetal and maternal circulations. However, that might mean that the relatively small changes in placental permeability from fetus to mother shown here might be more prominent in the human. The responses of these two species to an inflammatory event are similar with respect to the three plasma proteins AFP, transferrin and transthyretin (prealbumin); as in this study, these proteins have been reported to be acute phase negative proteins under inflammatory conditions ( Heinrich *et al.*, 1990; Hu *et al.*, 2019; Mackiewicz *et al.*, 1990). This supports the suggestion that these findings should be taken account of when advising pregnant women about the use of paracetamol. Given the unexpected findings of up-regulation of inflammatory cytokines and down-regulation of some acute phase plasma proteins, we are in the process of carrying out RNA-Seq replication studies and extending the range of cytokines estimated in fetal and maternal blood. Unfortunately, these experiments have been delayed by the COVID-19 emergency, which has closed our laboratories for an indefinite period. In view of the potential significance of our findings for the use of paracetamol in pregnancy, particularly the high frequency of its use, we feel it is justified to present these findings for peer review, in their present form.

### Clinical relevance

Paracetamol (acetaminophen) is generally considered “safe” to use in pregnancy and lactation ( Australian Medicines Handbook, 2019; Briggs *et al.*, 2017) although it is one of the most commonly overdosed drugs, including in pregnancy (Rayburn *et al.*, 1984). However, some authors urge caution in its use because of evidence of adverse effects ( Brune *et al.*, 2015). It has been reported that as many as nearly 80% of pregnant women in some populations ingest paracetamol ( Dreyer *et al.*, 2015). The findings of the present study, although based solely on experiments in rats, should be taken account of when advising pregnant patients on the use of paracetamol in pregnancy. The clinical situation is not straightforward because in addition to taking paracetamol to relieve pain, it may also be taken to reduce an increase in body temperature accompanying an infection (often respiratory), but there is evidence of an association between infection/fever and adverse outcomes for pregnancies; this seems to be a particular problem when infection/fever occurs at the beginning of the 3 ^rd^ trimester ( Hagberg *et al.*, 2015). Thus, continued but limited use of paracetamol to control severe pain and to reduce body temperature at critical stages of pregnancy would seems to be appropriate but not the widespread use for lesser indications that is implied by the reports that most pregnant women take paracetamol.

Increased transfer of sucrose and AFP from fetal circulation into maternal circulation, as demonstrated in the present study, suggests that other molecules/metabolites could potentially also reach the maternal circulation. There are several clinical implications, including that increased AFP levels detected in pregnant women are used to detect potential neural tube closure defects, although this test is done earlier in pregnancy and we have as yet no evidence of paracetamol affecting placental permeability this early in pregnancy.

Further investigation is required to see if there are similar effects in the placentas of patients who have taken paracetamol. If the effect is indeed confined to the fetal side of the placenta it will be clinically difficult to determine such an effect in pregnant patients, particularly if it turns out to be variable as in our rat experiments, although transfer of AFP from fetal to maternal circulation might be a useful indicator.

## Data availability

### Underlying data

RNA-Seq data on NCBI, Accession number PRJNA633629: https://identifiers.org/ncbi/bioproject:PRJNA633629

Figshare: Effects of paracetamol on rat placenta and fatal brain. https://doi.org/10.26188/5ebff4c2781a0 ( Koehn *et al.*, 2020)

This project contains the following underlying data:

- 200514 ELISA raw data.xlsx (raw data for the IL-1β ELISA )
- 200514 sucrose permeability data.xlsx (brain, CSF and plasma levels of sucrose in pregnant rats and fetuses)
- RA708 chronic high dose paracetamol.zip (plasma protein and H&E stained sections in Figure 10, A: RA708-50-04 HE x4.jpg, B: RA708-46-05 PP x4.jpg, C: RA708-46-05 PP x40.jpg
- RA677 actute high dose paracetamol.zip (plasma protein stained section in Figure 10, D: RA677-41-03 x40 B.jpg)
- 20191204 AFP loExp 1.tif (original unedited western blot image for Figure 8)

Data are available under the terms of the Creative Commons Attribution 4.0 International license (CC-BY 4.0).
