# Supplementary material for: Effects of paracetamol (acetaminophen) on gene expression and permeability properties of the rat placenta and fetal brain
Source: F1000Res. 2020 Aug 19;9:573. Originally published 2020 Jun 8. [Version 2] doi: 10.12688/f1000research.24119.2 (PMC7477648; doi:10.12688/f1000research.24119.2)
Supplement: Peer review history

# Peer review: Reviewer response for version 2

**DOI:** 10.5256/f1000research.28770.r69768

**Published:** 2020-09-07

**Authors:** Patrizia Ferretti

**Affiliations:** 1 Stem Cells and Regenerative Medicine Section, UCL Institute of Child Health, University College London, London, UK

I thank the authors for carefully considering my comments. I am happy with their replies, hence I endorse indexing of their interesting manuscript.

Is the work clearly and accurately presented and does it cite the current literature?

Partly

If applicable, is the statistical analysis and its interpretation appropriate?

Partly

Are all the source data underlying the results available to ensure full reproducibility?

Yes

Is the study design appropriate and is the work technically sound?

Yes

Are the conclusions drawn adequately supported by the results?

Partly

Are sufficient details of methods and analysis provided to allow replication by others?

Yes

Reviewer Expertise:

stem cells, tissue repair, developmental biology

I confirm that I have read this submission and believe that I have an appropriate level of expertise to confirm that it is of an acceptable scientific standard.

# Peer review: Reviewer response for version 1

**DOI:** 10.5256/f1000research.26605.r64436

**Published:** 2020-07-20

**Authors:** Patrizia Ferretti

**Affiliations:** 1 Stem Cells and Regenerative Medicine Section, UCL Institute of Child Health, University College London, London, UK

This paper addresses an important and neglected question of potential negative effects of paracetamol during pregnancy. It examines gene expression changes in placenta and foetal brain and also presents out a number of functional studies to establish whether placenta permeability or brain barriers in the fetus are affected by the treatment. The study reports significant changes in the expression of a number of genes, including genes associated with the immune response, and validates changes in one of these genes, Il1ß, at the protein level in the placenta. While in some respects this study is still preliminary, the information presented here is valuable for underpinning future studies.

 The authors clearly explain their choice of the end point (E19) selected, but not the reason for starting the chronic treatment at E15, rather than earlier, when important developmental events occur and teratogenic effects might be more likely and significant.

 Specific Comments:

 P6: It is not clear why only the t-test was used when comparing multiple groups, as ANOVA followed by a post-hoc test should have been used.

 On p7 the authors say “...65 up-regulated and 57 down-regulated....”, but Fig. 1 indicates 64 up-regulated genes, consistent wih the total of 121 up and down-regulated transcripts indicated in the right column.

 The authors indicate that expression of 737 genes is significantly affected by chronic treatment, but do not show the level of significance. Does this mean that p is <0.05 (but never <0.01 or smaller) for all transcripts?

 Table 1 and 3. It would be helpful to colour code genes that change in both acute and chronic treatment groups and use thicker vertical lines between groups for ease of visualization.

 Table 2 includes genes that are not in the top 50 shown in Table 1, and this should be clearly stated (at a first glance the Table seemed a bit redundant). As for Table 1, the level of significance should be indicated. The Table could be made it easier to read if the “up-regulated (acute/control)” genes were shown below the “up-regulated (chronic/control)”, rather than in adjacent columns, or were clearly separated using a thicker vertical line. In addition, it is confusing to have a column “chronic/control” under the “up-regulated (acute/control)” list. This seems to have been done to accommodate S100a rather than inserting it under each comparison. Please check carefully that the difference indicated in different Tables are the same (e.g. S100a8 has a FC 2.25 in Tab1 and FC 2.26 in Table 2). “Il1b” should be changed to “Il1ß”. A pie chart of the inflammatory genes to complement Table 2 and Fig. 3 would be useful.

 P17, left column, top and Fig. 4. There is clearly variability, but to give numbers of fetuses where Il1ß levels could be detected over total numbers assayed for all groups would be more accurate and informative (e.g. acute 2/4, chronic low 7/16 and chronic high 10/19) than including these number only for the chronic high group, which appears to be wrongly given as 19/39, while the number of fetuses indicated for this group in Fig. 5 legend is 19.

 Figs 6 and 7 do not include error bars and no statistical analysis of these data seems to have been performed. It should be clearly indicated whether there was no statistical difference among groups at any time point studied.

 P20, left column, top. The statement: “AFP levels were higher in all treated dams compared to an un-treated control.” should be revised, as Fig. 8 shows an AFP increase only in chronically treated dams. It is a pity that the number of dams is too small to assess the significance of this observation and that no housekeeping protein was used to normalize AFP expression. If B is a densitometry of the gel in A, where according to the western blot labelling and the legend there is only 1 control for both dam and fetus, why are there 2 samples indicated in the controls in the charts? Given the low sample numbers and variability, particularly in fetal AFP levels, expressing the data as ratio is not appropriate.

Is the work clearly and accurately presented and does it cite the current literature?

Partly

If applicable, is the statistical analysis and its interpretation appropriate?

Partly

Are all the source data underlying the results available to ensure full reproducibility?

Yes

Is the study design appropriate and is the work technically sound?

Yes

Are the conclusions drawn adequately supported by the results?

Partly

Are sufficient details of methods and analysis provided to allow replication by others?

Yes

Reviewer Expertise:

stem cells, tissue repair, developmental biology

I confirm that I have read this submission and believe that I have an appropriate level of expertise to confirm that it is of an acceptable scientific standard, however I have significant reservations, as outlined above.

# Response

**Published:** 2020-08-02

**Authors:** Norman Saunders

**Affiliations:** Department of Pharmacology and Therapeutics, University of Melbourne, Australia

*We should like to thank Professor Ferretti for her detailed review of this paper and in particular for her helpful suggestions for clarification and improvement of presentation of the results of this study. We provide responses below to all of the matters raised by Professor Ferretti.*

 This paper addresses an important and neglected question of potential negative effects of paracetamol during pregnancy. It examines gene expression changes in placenta and foetal brain and also presents out a number of functional studies to establish whether placenta permeability or brain barriers in the fetus are affected by the treatment. The study reports significant changes in the expression of a number of genes, including genes associated with the immune response, and validates changes in one of these genes, Il1ß, at the protein level in the placenta. While in some respects this study is still preliminary, the information presented here is valuable for underpinning future studies.

 The authors clearly explain their choice of the end point (E19) selected, but not the reason for starting the chronic treatment at E15, rather than earlier, when important developmental events occur and teratogenic effects might be more likely and significant.

*The chronic treatment was limited to the last week of pregnancy in the rats, starting at E15, because it involved twice daily intraperitoneal (IP) injections of the drug and we were concerned that a longer treatment was an unreasonable imposition on the animals. In addition, it is well known that pregnant rats, if unduly stressed, are prone to aborting their fetuses. Also as mentioned in the 1 ^st^ paragraph of the Discussion, the period of treatment covers about 25% of the gestational period of the rat, which is the main period that we are currently interested in. Oral medication would have been an alternative but it gives much less control over the amount of drug administered than when administered by IP injection. To have any control of oral administration requires monitoring of blood levels of a drug (itself an invasive process). This was not available at the time of this study but has now been developed for future studies. In relation to possible teratogenic effects, this was not an aim of the study and would, as the Reviewer indicates, require administration earlier in pregnancy. There is only limited information on possible teratogenic effects of paracetamol, probably because it came into clinical use long before specific regulatory requirements (e.g. FDA) for teratogenic testing in animals came into force.*

*Studies involving limited epidemiological data have concluded that there is no evidence of an association between paracetamol ingestion and congenital malformations (Briggs et al. 2019, pp8-11).*

 Specific Comments:

 P6: It is not clear why only the t-test was used when comparing multiple groups, as ANOVA followed by a post-hoc test should have been used.

*For the analysis of RNA-Seq data, the t-test is part of the packages we used and includes built in posthoc corrections for multiple comparisons. For the data on Il1ß the advice we have from our departmental statistical expert is that t-tests are appropriate for this type of research: (Lew M.J. (2019) A Reckless Guide to P-values. In: Bespalov A., Michel M., Steckler T. (eds) Good Research Practice in Non-Clinical Pharmacology and Biomedicine. Handbook of Experimental Pharmacology, vol 257. Springer, Cham.).*

*Nevertheless in view of the Reviewer’s comment we have run the data through ANOVA*

*followed by Tukey's posthoc test. The significance levels are the same as those we obtained with a t-test. We have added this information to the Methods subsection “Statistics”.*

 On p7 the authors say “...65 up-regulated and 57 down-regulated....”, but Fig. 1 indicates 64 up-regulated genes, consistent with the total of 121 up and down-regulated transcripts indicated in the right column.

*We thank the Reviewer for drawing our attention to this error which has been corrected.*

 The authors indicate that expression of 737 genes is significantly affected by chronic treatment, but do not show the level of significance. Does this mean that p is <0.05 (but never <0.01 or smaller) for all transcripts?

*As indicated in the Methods section on “Statistical Analysis” we used P<0.05 for two of the three analyses used. We focussed on genes with large fold changes as these are more likely to be of functional significance than would be indicated by a higher level of statistical significance.*

 Table 1 and 3. It would be helpful to colour code genes that change in both acute and chronic treatment groups and use thicker vertical lines between groups for ease of visualization.

*We thank the Reviewer for this suggestion. The Tables have been modified accordingly. The treatment groups are now separated by a gap. The colour coding highlights some interesting differences in the number of genes that responded in the different treatment groups. A note of this has been added to the Table legends and in the text.*

 Table 2 includes genes that are not in the top 50 shown in Table 1, and this should be clearly stated (at a first glance the Table seemed a bit redundant).

*This Table shows only inflammatory and immune-related genes and therefore some genes in the top 50 in Table 1 do not appear here. This is now indicated in the legend.*

 As for Table 1, the level of significance should be indicated.

*P<0.05 added to legend.*

 The Table could be made it easier to read if the “up-regulated (acute/control)” genes were shown below the “up-regulated (chronic/control)”, rather than in adjacent columns, or were clearly separated using a thicker vertical line.

*This change would make a very long 2 column table. We prefer the helpful suggestion that the columns should be separated which we have done with a narrow blank column*

 In addition, it is confusing to have a column “chronic/control” under the “up-regulated (acute/control)” list. This seems to have been done to accommodate S100a rather than inserting it under each comparison.

*Unfortunately in the editorial process of preparing the pdf from the submitted Table spreadsheets some of the down-regulated genes have been sliced off and put incorrectly under the up-regulated categories. We are puzzled by this as the proof we received to check was correct. I have discussed this with the Editorial Office who have indicated that they will make sure this does not occur in the next version.*

 Please check carefully that the difference indicated in different Tables are the same (e.g. S100a8 has a FC 2.25 in Table1 and FC 2.26 in Table 2).

*This was due to a difference in rounding, which has now been corrected.*

 “Il1b” should be changed to “Il1ß”.

*Il1b is the notation used in the gene data base ncbi.nim.nih.gov,*

*we would prefer to retain this notation in tables.*

 A pie chart of the inflammatory genes to complement Table 2 and Fig. 3 would be useful.

*We generally find that pie charts are not helpful and would prefer not to make this addition.*

 P17, left column, top and Fig. 4. There is clearly variability, but to give numbers of fetuses where Il1ß levels could be detected over total numbers assayed for all groups would be more accurate and informative (e.g. acute 2/4, chronic low 7/16 and chronic high 10/19) than including these number only for the chronic high group, which appears to be wrongly given as 19/39, while the number of fetuses indicated for this group in Fig. 5 legend is 19.

*We think that the Reviewer is probably referring to Fig 5. We agree that the way of representing these data that the Reviewer has suggested is clearer. This has been incorporated into the text (bottom P30). Figure 5 has been modified to make it clearer that values were obtained from 4 control fetuses. The legend has been re-written to make it clearer how many dams and fetuses were involved in this part of the study.*

 Figs 6 and 7 do not include error bars and no statistical analysis of these data seems to have been performed. It should be clearly indicated whether there was no statistical difference among groups at any time point studied.

*Each point is a single fetus. The n values represent the number of fetuses in each treatment group. The legend has been rewritten to explain this more clearly.*

 P20, left column, top. The statement: “AFP levels were higher in all treated dams compared to an un-treated control.” should be revised, as Fig. 8 shows an AFP increase only in chronically treated dams.

*This has been revised to state that there was an increase in dams’ AFP only in the chronically treated animals.*

 It is a pity that the number of dams is too small to assess the significance of this observation and that no housekeeping protein was used to normalize AFP expression. If B is a densitometry of the gel in A, where according to the western blot labelling and the legend there is only 1 control for both dam and fetus, why are there 2 samples indicated in the controls in the charts? Given the low sample numbers and variability, particularly in fetal AFP levels, expressing the data as ratio is not appropriate.

*We agree that it is a pity that the numbers were very small, but we were constrained by the effects of being shut out of our laboratories for several months because of the coronavirus emergency. In general the only way to obtain accurate AFP values is to measure the actual concentrations of the protein. We are very aware that Western blots are only semi-quantitative at best. We attempted to make the gels from which we took measurements as comparable as possible within each age group by using similar volumes of plasma (or diluted sample). The concentrations of plasma proteins vary between different animals and are not related to each other therefore using albumin as a reference protein would not provide more clarity. We thank the reviewer for drawing our attention to the discrepancy in control adult numbers in the western blots (A) and in the densitometry readings (B). This has been corrected.*

# Peer review: Reviewer response for version 1

**DOI:** 10.5256/f1000research.26605.r64439

**Published:** 2020-07-07

**Authors:** Roland Bainton

**Affiliations:** 1 Department of Anesthesia and Perioperative Care, University of California, San Francisco (UCSF), San Francisco, CA, USA

**Overview.**

 The authors present a well-controlled immaculately-conceived and artfully interpreted paper on proteomic and genomic responses of the mammalian placenta to the most commonly administered drug in the world, paracetemol.

 The results of this paper are quite profound and long overdue. There are few are very few studies that attempt as complete an evaluation of drug response and in an organ specific manner. This authorship team has exploited the most pertinent drug interfaces for chemoprotection of the fetus to glimpse the system of toxicologicologic protection of the developing fetus and brain. They exploit two well known pharmacologically highly regulated barrier interfaces: the placenta and BBB. As experts on barrier development they have the right expertise to measure the developmental role and robustness of these understudied barrier interfaces to the drug paracetemol. Tylenol (as known by the US brand name) is a ubiquitous pharmacologic agent used world-wide for the abrogation of pain and systemic suppression of inflammation. While deemed one of the safest medications ever invented because of its common utilization by every age group and gender, and its long standing well-described clinical toxicities suggests that it has been vetted for safety over and over. But with the right question and under the correct experimental circumstance profound novel sensitivities in the physiology of mammals can be discovered. Such is the insight of this manuscript.

**Of note.**

 The paper is very complete. They demonstrate both acute and chronic changes to the placenta transcriptome with strong statistical relevance. Interestingly the chronic and acute genetic changes have few if any overlapping genes suggesting that long term toxicologic homeostasis may have very different effects to fetal development than single dosing. Thus, as noted by the authors, the use for the control of acute inflammatory responses may be warranted, but chronic ingestion any substance should be view with caution when the developing fetus is concerned.

**Conclusion.**

 This paper is well conceived, clearly written and expertly interpreted. Safety profiles of drugs are in flux and whether vertebrate homeostatic metabolic responses to drug exposure, acute or chronic, is truly benign is an open question. These authors clearly demonstrate, by the discovery of soluble protein changes in dosing of paracetamol, that there is more to learn about drug toxicology, in particular at the chemoprotective interfaces of the body, in this case the placenta and BBB. While the consequences of these proteomic changes are unclear they are corroborated by profound compensations in the transcriptional profiles of the placenta. Interestingly, the placental barrier does the lion share of compensation as the BBB of pups is nearly unchanged. This is a reassuring finding for the developing brain, but leaves many unanswered questions about how the fetus may affect maternal physiology (as noted by the authors). 

 The implications of this study are profound and not only for the use of paracetamol. In this paper they describe a road map for the study of all drugs that could have maternal fetal interactions and provide the physiologic and genomic insights to back up their assertions. Indeed their proposed experiments in pregnant women to follow up on their findings would be very important to the management of pregnancy and to the field of maternal/fetal physiology as a whole.

 I love this paper. BRAVO!

 Major Issues

 None.

 Minor Issues.

 None.

 Typos.

 None. 

Is the work clearly and accurately presented and does it cite the current literature?

Yes

If applicable, is the statistical analysis and its interpretation appropriate?

Yes

Are all the source data underlying the results available to ensure full reproducibility?

Yes

Is the study design appropriate and is the work technically sound?

Yes

Are the conclusions drawn adequately supported by the results?

Yes

Are sufficient details of methods and analysis provided to allow replication by others?

Yes

Reviewer Expertise:

Blood brain barrier, genomic toxicology, biomarkers of stress and tissue injury, blood diagnostics, metabolic compensations of the CNS

I confirm that I have read this submission and believe that I have an appropriate level of expertise to confirm that it is of an acceptable scientific standard.

# Response

**Published:** 2020-08-03

**Authors:** Norman Saunders

**Affiliations:** Department of Pharmacology and Therapeutics, University of Melbourne, Australia

We thank Dr Bainton for his detailed and perceptive commentary on our study of paracetamol/acetaminophen in pregnancy and in the newborn period. We particularly appreciate his view that our findings raise serious concerns about the use of this drug in pregnancy. At this stage we have only animal data that raises concerns, but we plan to follow up with human studies insofar as this is possible. We hope that our findings will give pause for thought by the regulatory authorities and doctors who regard paracetamol/acetaminophen as “safe” to be used in pregnancy and breast feeding, especially as the concept of “being safe” for any drug is a dubious one, and particularly for one that is used so frequently. We also appreciate the Reviewer’s comment that our approach provides a “roadmap” for studies of the many drugs that are prescribed in pregnancy about which there is little or no evidence on entry  across the placenta and into the fetal brain. We are currently undertaking studies of psychotropic and anti-epileptic drugs as well new drugs introduced for the treatment of cystic fibrosis. Of course, the best outcome would be to find that little or no drug crosses the placenta and enters into the fetal brain.

 Dr Bainton’s comments are very important in helping to maintain this type of *in vivo* study.
